# Diverse integrated ecosystem approach overcomes pandemic-related fisheries monitoring challenges

**DOI:** 10.1038/s41467-021-26484-5

**Published:** 2021-11-11

**Authors:** Jarrod A. Santora, Tanya L. Rogers, Megan A. Cimino, Keith M. Sakuma, Keith D. Hanson, E. J. Dick, Jaime Jahncke, Pete Warzybok, John C. Field

**Affiliations:** 1 Fisheries Ecology Division, Southwest Fisheries Science Center, National Marine Fisheries Service, National Oceanic and Atmospheric Administration, Santa Cruz, California 95060 USA; 2 Department of Applied Math, University of California Santa Cruz, Santa Cruz, California 95060 US; 3 Institute of Marine Sciences, University of California Santa Cruz, Santa Cruz, California 95060 USA; 4 Environmental Research Division, Southwest Fisheries Science Center, National Marine Fisheries Service, National Oceanic and Atmospheric Administration, Monterey, California 93940 USA; 5 Point Blue Conservation Science, Petaluma, California 94954 US

**Keywords:** Fisheries, Ecosystem ecology, Ecosystem ecology

## Abstract

The COVID-19 pandemic caused unprecedented cancellations of fisheries and ecosystem-assessment surveys, resulting in a recession of observations needed for management and conservation globally. This unavoidable reduction of survey data poses challenges for informing biodiversity and ecosystem functioning, developing future stock assessments of harvested species, and providing strategic advice for ecosystem-based management. We present a diversified framework involving integration of monitoring data with empirical models and simulations to inform ecosystem status within the California Current Large Marine Ecosystem. We augment trawl observations collected from a limited fisheries survey with survey effort reduction simulations, use of seabird diets as indicators of fish abundance, and krill species distribution modeling trained on past observations. This diversified approach allows for evaluation of ecosystem status during data-poor situations, especially during the COVID-19 era. The challenges to ecosystem monitoring imposed by the pandemic may be overcome by preparing for unexpected effort reduction, linking disparate ecosystem indicators, and applying new species modeling techniques.

## Abstract (web summary)

The Covid-19 pandemic has disrupted ecosystem and biodiversity monitoring programs, including marine fisheries surveys. Here the authors combine multiple modelling approaches and data to overcome lost observational effort off the coasts of California in a diversified integrated ecosystem approach.

## Introduction

The coronavirus disease 2019 (COVID-19) pandemic resulted in extensive cancellations of marine fishery and ecosystem surveys conducted globally, causing a reduction in observational data^1–4^. The rapid increase in disease cases in the first quarter of 2020, along with lack of testing capacity and existing protocols, resulted in the decision to suspend nearly all US large fishery-independent research surveys until best safe practices were established^3–5^. This data loss poses new significant challenges for ecosystem science and the development of future fishery stock and ecosystem assessments that are used to manage and inform decisions about living marine resources. Furthermore, increased climate change and variability is impacting coastal ocean ecosystems^6,7^ and the loss of ecosystem monitoring data could make it more difficult to interpret interannual changes in fished resources and conservation of protected species^8,9^. Therefore, tools developed from decades of ecosystem monitoring may help fill information gaps and inform ecosystem condition during a time of little to no data collection^10^. In this study, we investigate a diverse suite of analytical tools for bridging the pandemic-driven recession of marine ecosystem observations. We explore ways to integrate knowledge of auxiliary data sources and forecasting models, and statistical resampling of past data, to generate a better characterization of ecosystem state (and uncertainty about this state) in years with reduced sampling.

Consideration of the impacts of unavoidable survey effort reduction is an increasingly high priority for marine resource management agencies worldwide, as research budgets shrink or hold constant and the costs associated with such surveys increase^11–13^. Perhaps serendipitously, the most recent effort to consider strategies for adapting to such impacts was held at the earliest stages of the 2020 pandemic^14^. This workshop (planned before the pandemic) summarized strategies for adaptation based on the latest science, including simulated tradeoffs between survey effort and the accuracy of survey indices, sampling design considerations, decision trees to inform decision-making regarding funding and ship time allocations, and advancing simulation and model-based capabilities to inform decision-making. We applied these diverse tools to investigate observation limitations caused by the pandemic on a representative fisheries survey.

**Fig. 1:**
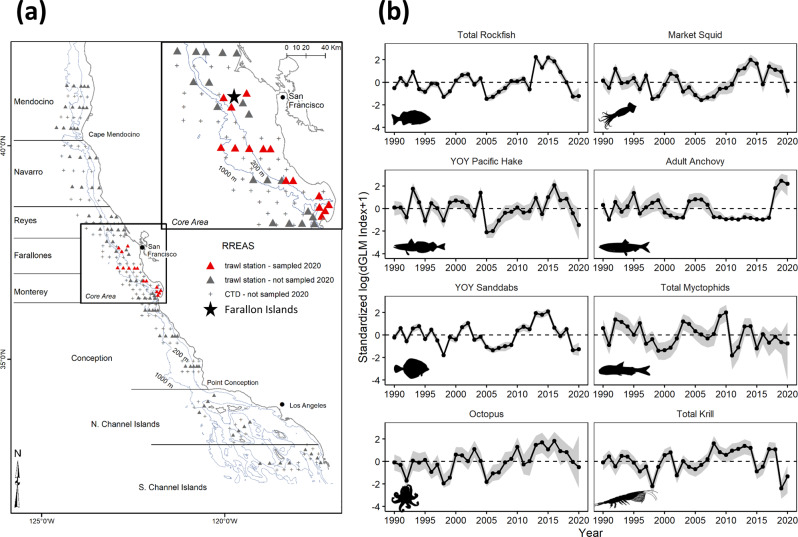
Fisheries and ecosystem-assessment survey. **a** US West Coast and study area of the Rockfish Recruitment and Ecosystem Assessment Survey. Distribution of mid-water trawl and oceanographic sampling stations; inset core area showing stations sampled in 2020 (red) and location of seabird monitoring studies on southeast Farallon Island. **b** Standardized model-based estimates of forage taxa that are used for assessment of annual ecosystem state and future recruitment patterns; YOY is “young-of-the-year.” Error bands (shaded area) are 95% credible intervals. Map made in ArcGIS. Taxa silhouettes are derived from phylopic.org.

The Rockfish Recruitment and Ecosystem Assessment Survey (RREAS) is an annual mid-water trawl survey that informs stock assessments and ecosystem status within the California Current Large Marine Ecosystem (CCLME). Observations and models derived from the RREAS are used to assess recruitment patterns of groundfish, abundance and distribution of epipelagic forage species, and ocean-climate processes influencing biodiversity and ecosystem functioning^15,16^. Ecosystem indicators derived from the RREAS provide reference points to resource managers, to benefit decisions regarding impacts of forage taxa, ranging from salmon populations to mortality events of seabirds and mammals. The survey is also a key node in the US Marine Biodiversity Observation Network and informs the health and status of biodiversity conditions within several National Marine Sanctuaries (Supplementary Fig. 1). By anticipating the loss of oceanographic vessel time for the 2020 survey (i.e., March 2020 lockdown), a range of biological collections were explored, including collecting a limited amount of mid-water trawls by contracting a commercial fishing vessel, other fishery-independent data known to covary with trawl-based observations (seabird diets) through partnership with regional scientific programs, and model-based approaches to estimating key species dynamics^17,18^. The survey was able to successfully sample ~25% of its normal trawl survey effort within its long-term study region (Fig. 1 and Supplementary Fig. 1), allowing continuation of a 38-year time series. The curtailed survey resulted in a natural stress test to assess impacts of unavoidable survey effort reduction due to the pandemic and the utility of available tools for mitigating this data loss.

We investigate how lost observational effort can be overcome through a diversified integrated ecosystem approach. Using several modeling approaches, we demonstrate how we updated ecosystem indicators using limited data and evaluated their robustness so as to maintain confidence in the trends and variability of key ecosystem components. We applied models that better account for spatially biased sampling and used resampling of past data to estimate changes in the accuracy and precision of estimates given observed (and potential) loss-of-survey effort^13,14,19^. We developed new models, trained on past survey data, including seabird diet observations, to evaluate abundance of young-of-the-year (YOY) rockfish (*Sebastes* spp.) and adult northern anchovy (*Engraulis mordax*), and a species distribution model (SDM) for monitoring status of krill (Euphausiidae) populations^17,18,20^. Knowledge of species interactions and ocean-climate conditions allowed us to parameterize these predictive models and produce forecasts using available and auxiliary data sources. This diversified approach can either boost confidence or highlight uncertainty in estimates from reduced sampling. Using this combination of methods allowed us to provide timely metrics of relative regional abundance of anchovy and incoming rockfish year classes to managers during this time period, although retrospective analyses (e.g., stock assessments that rely on demographic data from other surveys) will eventually provide additional information and context on the robustness of these results. These types of approaches can be easily extended to other ecosystem surveys, where such data and tools exist, and can inform the communication of uncertainty to fishery management councils, marine sanctuaries, and protected areas with regards to ecosystem status and trends during the COVID-19 era^4,9,21^.

## Results and discussion

### Conducting an ecosystem survey during a pandemic

Cancellation of the survey aboard its primary National Oceanic and Atmospheric Administration (NOAA) survey vessel was overcome through acquisition of a charter for a commercial fishing vessel, following all COVID-19 guidelines (Supplementary Figs. 1 and 2). Initial plans were for 15 days at sea, rather than the 45 typically conducted. This lower effort, along with adverse weather and vessel constraints, resulted in only 25% of the average number of mid-water trawls being collected in the long-term core survey area (Fig. 1 and Supplementary Fig. 1). Despite the data reduction, this effort was one of the only fisheries independent surveys to occur on the US West Coast after the first lockdown in March 2020, furthering the need to evaluate impacts of reduced sampling and provide a robust synthesis of survey results for fishery management. Here we provide updated indices for a selection of ecologically and commercially important species that are critical for assessing ecosystem status.

**Fig. 2:**
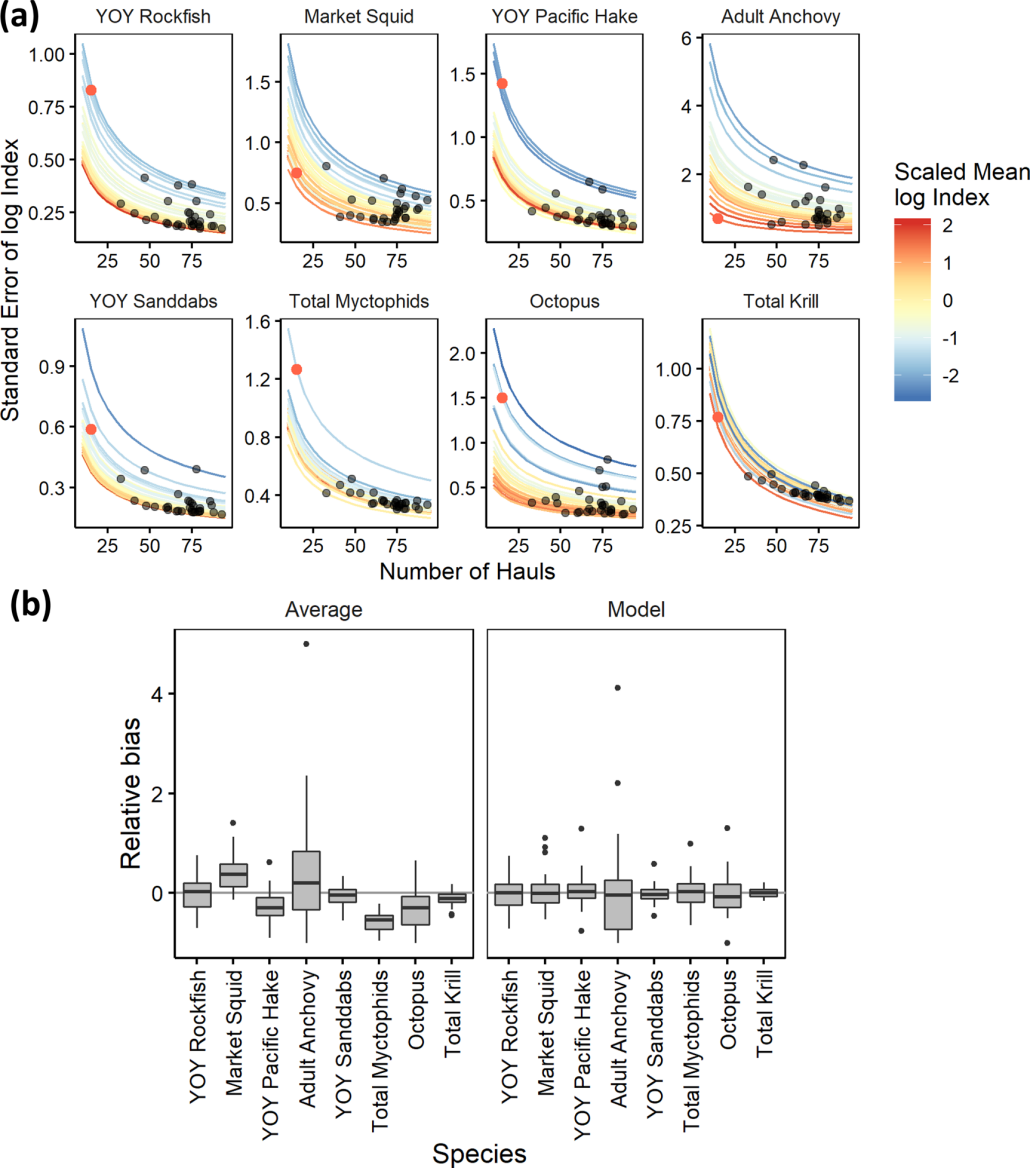
A model for uncertainty and unavoidable effort reduction. **a** SE of log index vs. number of hauls for a given year from the delta-GLM model. Each point is a year, with 2020 indicated in red. Lines are predicted relationship between SE and sample size for each year, color indicating the mean log index for that year, scaled within taxa. **b** Relative bias in the index point estimate using 15 hauls from the 2020 stations vs. all hauls from all stations sampled in a given year, computed as (*x*_2020_ − *x*_all_)/x_all_. Boxplots show spread of results across all years, 1990–2019 (*n* = 30 independent years, center: median, box: first and third quartiles, whiskers: smallest and largest values no further than 1.5× IQR from the first and third quartiles; IQR, interquartile range). In the left panel, the index was computed by averaging values of log(CPUE + 1) from all available hauls in a given year. In the right panel, the index was computed from the maximum likelihood estimate (MLE) of a delta-GLM model with spatial covariates, as log(MLE + 1). For the model-based index, the *x*_2020_ estimate excludes hauls from the focal year but includes complete data from all other years. CPUE, catch-per-unit-effort; GLM, generalized linear model.

The 2020 sampling was spatially biased towards inshore (shallow) stations (Fig. 1) and thus the previously used method for calculating abundance indices (averaging log-transformed catch-per-unit-effort (CPUE), across all sampled stations) was expected to result in biased indices, in particular for species with strong nearshore (e.g., market squid *Dorytheuthis opalescens*, anchovy) or offshore (YOY Pacific hake *Merluccius productus*, myctophids Myctophidae, octopus Octopoda, krill) habitat associations (Supplementary Fig. 3). We confirmed that this bias does indeed occur by recomputing indices for the past 30 years, but using only 1 trawl from each of the 15 stations that were sampled in 2020, and comparing these indices to those using all available trawls (Fig. 2 and Supplementary Fig. 4). In contrast, model-based indices computed from equivalently subsampled past data did not show systematic bias due to the incorporation of spatial covariates (Fig. 2). Thus, although the average log CPUEs were well correlated with model-based indices for well-sampled years (1990–2019), average log CPUEs were determined to be inappropriate for 2020 reporting, and the model-based results were used to develop indices for all taxa for years 1990–2020.

The 2020 model-based indices for total rockfish and sanddab (*Citharichthys* spp.) were the second lowest on record and continued a decline from record high abundance levels observed during the 2014–2016 marine heatwave (Fig. 1)^22,23^. Pacific hake, myctophids, and octopus were also below average. In contrast, the 2020 index for adult northern anchovy continued a multi-year period of persistently high abundance (Fig. 1). Market squid indices were below average, following a mostly positive trend over the past 7 years. Following the steep decline in 2019, the krill index in 2020 was lower than average (Fig. 1); however, as discussed below, uncertainty may be underestimated for this highly patchy taxonomic group. As a consequence of the low sample sizes, a more rigorous evaluation of the trade-off between sample size (trawls) and uncertainty was conducted, as well as further evaluation of trends through application of existing ecosystem science tools.

### Quantifying uncertainty by resampling the past

For most taxa, the uncertainty associated with the 2020 relative abundance estimate was the greatest in the time series, an intuitive result of the sparse sampling for that year (Figs. 1b and 2). The SE was estimated to be over three times the long-term average SE for rockfish and Pacific hake, myctophids, and octopus, and the largest (but less than double the long-term mean) for sanddabs and krill (Fig. 2a). By contrast, the uncertainty associated with the adult anchovy index was lower than the long-term average, due to the great abundance and high frequency of occurrence of anchovy in 2020, compared to years in past decades. This reflects the general trend of uncertainty (on the log scale) being greater for a given taxon when abundance is lower, which generally held for all taxa except krill in our explorations (Fig. 2 and Supplementary Fig. 5). Through time, the relative bias of the subset of stations (2020) vs. the full sample size is also consistently lower for the model-based solution compared to using the average estimate (Fig. 2 and Supplementary Fig. 6). There is also a strong relationship between the number of trawls conducted and the resulting error for each point estimate, with the error essentially doubling when the number of trawls is reduced from the long-term average of 62 to the 15 that were conducted in 2020 (Fig. 2a). By contrast, reducing the total number of trawls from 62 to 40 increases the relative error by just under 25%, while increasing the number of trawls from 62 to 90 only decreases the relative error by 16%. The extent to which the mean relative abundance scales that error up or down, regardless of sample size, is taxon specific. There is an approximate doubling of the error at lowest abundance levels relative to the highest levels for rockfish, sanddabs, hake, and market squid, an increase of more than fourfold over the same range for anchovies and octopus, and relatively modest scaling of the error for myctophids and krill (Fig. 2). This trade-off between survey effort and the error of the ecosystem indices provides critical guidance for future survey planning with respect to the complex trade-off between effort and uncertainty in the face of highly variable interannual catch rates.

### A seabird’s perspective

**Fig. 3:**
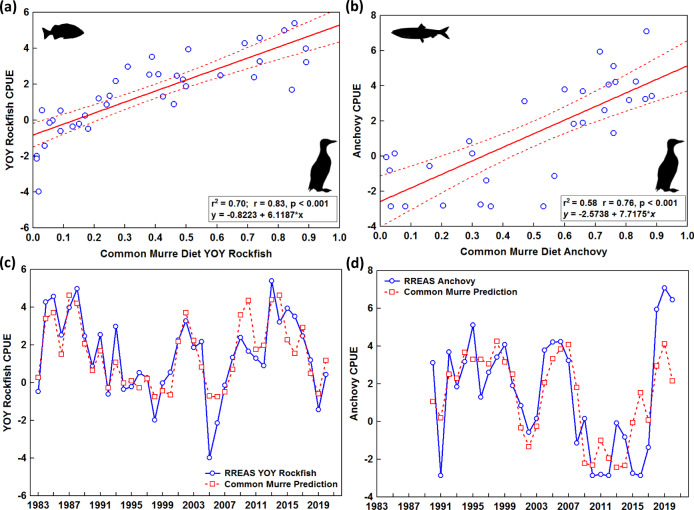
A seabird diet and ecosystem indicator model. **a** Functional relationship between YOY Rockfish CPUE (catch-per-unit-effort) log abundance index and mean proportion of YOY rockfish in seabird (common murre) diet; 1983–2019. **b** Functional relationship between adult northern anchovy CPUE log abundance index and mean proportion of anchovy in seabird (common murre) diet; 1990–2019. Dashed lines in **a**, **b** are 95% confidence intervals. **c**, **d** Prediction of YOY Rockfish and anchovy abundance index from the seabird regression model (dashed line) compared to log(CPUE) from the delta-GLM (points). Taxa silhouettes are derived from phylopic.org.

The Farallon Islands (National Wildlife Refuge) are located in the center of the survey region and host the largest breeding colony of common murre (*Uria aalge*) in the region (Fig. 1). Interannual variability of Farallon Island seabird population dynamics, reproduction, and foraging ecology are well understood and also track RREAS observations^6,17^. In particular, patterns such as alternating cycles of forage species occurrence and subsequent reproductive output are known to be linked to regional ocean and climate conditions^17,20^. Long-term observations of seabird diets in the Farallon Islands were fortunately not impacted by the pandemic. As common murre feed their chicks predominantly either juvenile rockfish or northern anchovy (Supplementary Fig. 7), and common murre prey selection is known to covary with prey abundance in the surrounding ecosystem^17,20^, these observations provide a critical data stream for evaluating 2020 rockfish and anchovy abundance index estimates from the limited trawl sampling. We updated regression models relating the proportion of rockfish and anchovy in murre diets, respectively, to model-based abundance indices for rockfish and anchovy using past data (Fig. 3). Linear models provided the best fit for YOY rockfish and anchovy, (*r*^2^ = 0.70; *r*^2^ = 0.58, respectively, both *p* < 0.001). During 2020, common murre diet was mixed, with 33% rockfish and 61% anchovy (Supplementary Fig. 7). Application of the seabird regression model produced 2020 index predictions that were largely in agreement with the 2020 indices generated from limited trawl data. From the common murre’s perspective, rockfish was slightly higher and anchovy was slightly lower than suggested by the trawl survey, but estimates were within the 95% confidence intervals. This new seabird tool can be applied in the event of future survey cancellations and time series estimates can be used in stock assessment and food-web studies. Many seabird population and diet data sets are available throughout the world^24^ and effort should be made to derive similar models with fishery-independent data sets. Further, data streams such as seabird diets could be incorporated directly into multi-observation models, e.g., fish abundance modeled as a latent variable sampled by multiple observation processes (i.e., trawls and birds) in a Bayesian framework^25^. This diversified data integration approach should allow for more robust estimates and strengths in one data stream may make up for deficiencies in another.

### Krill species distribution modeling

**Fig. 4:**
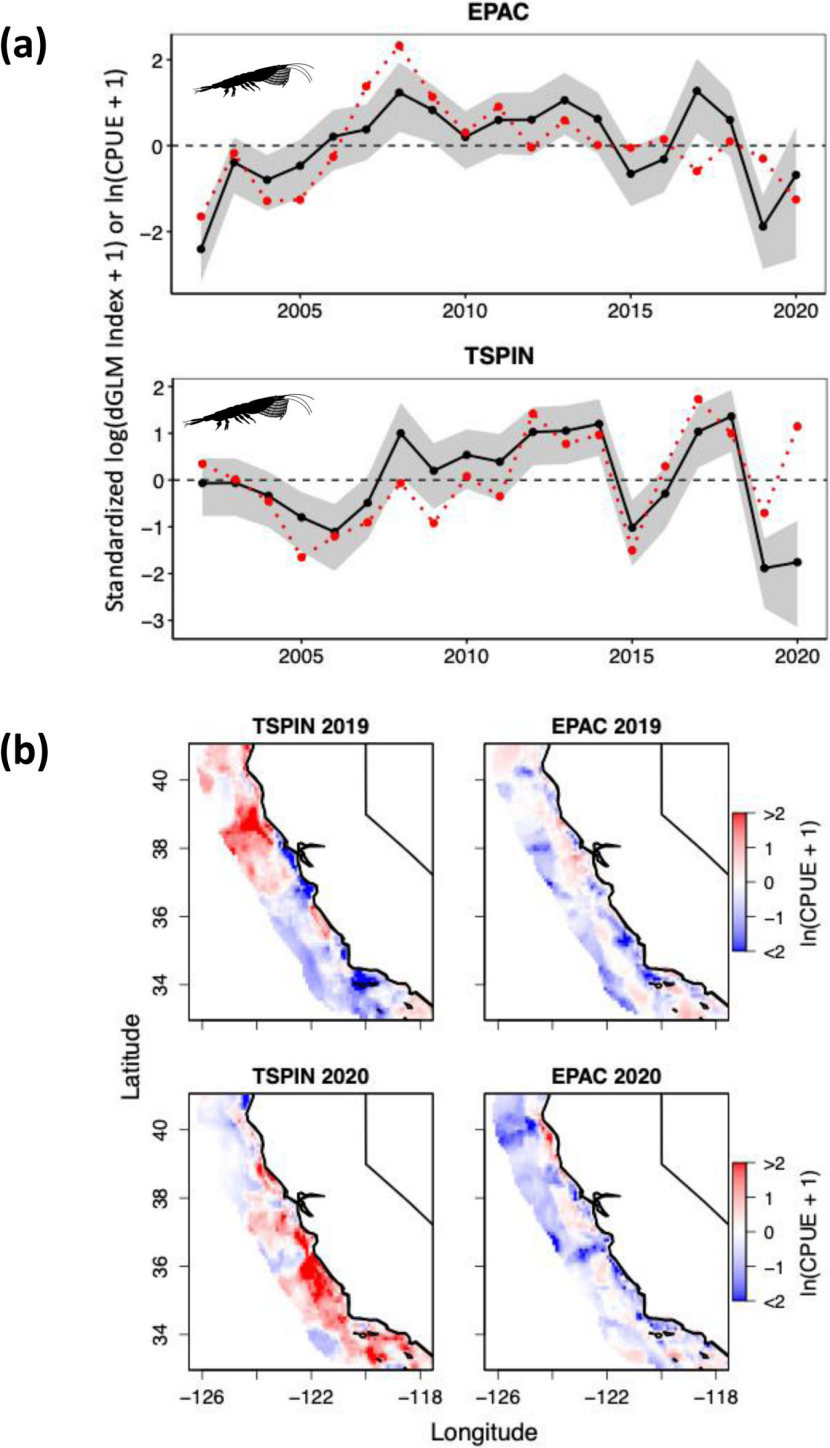
Prediction of krill species distribution and abundance. **a** Interannual variation in standardized log(delta-GLM Index + 1) estimates (black line) and species distribution model (SDM) mean ln(CPUE + 1) (red dashed line) for *T. spinifera* (TSPIN) and *E. pacifica* (EPAC) from 2002 to 2020 within the core region. CPUE, catch-per-unit-effort. Error bands (shaded area) are 95% credible intervals. It is noteworthy that observations from the 2020 trawl survey are likely to be underestimated. **b** Spatial anomalies of predicted TSPIN and EPAC abundance from the mean CPUE climatology from 2002 to 2018 during 2019 and 2020. Red (blue) indicates higher (lower) than average CPUE and only predictions out to 150 km from shore are shown. See Supplementary Fig. 9 for mapped comparisons between predictions across this domain and station-level observations. Taxa silhouettes are derived from phylopic.org.

Relative abundance of krill is a critical ecosystem indicator that is used to monitor the health and functioning of the coastal and offshore marine food webs^9,26,27^. Due to their high abundance and tendency to form dense aggregations (hotspots), reduced offshore sampling likely impacts the assessment of overall krill abundance and regional distribution patterns in 2020 (Fig. 1 and Supplementary Fig. 8). Application of SDMs that are parameterized and trained on historical, environmental, and biological observations are potentially important tools for predicting krill species abundance during reduced sampling (Fig. 4 and Supplementary Fig. 9). The 2020 indices were highly uncertain due to limited sampling, so we applied the delta-generalized linear model (GLM) approach and the new krill SDM to predict relative abundance^18^.

Although the delta-GLM index from the trawl survey suggested that total krill abundance in 2020 continued to be low following a steep decline in 2019 (Fig. 1b and Supplementary Fig. 7), the SDMs revealed different patterns for the two dominant krill species (Fig. 4). Relative to long-term averages, the SDM approach indicated that the coastal species, *Thysanoessa spinifera*, was higher in abundance, whereas *Euphausia pacifica*, the numerically dominant and offshore species, was lower than average (Fig. 4a). The delta-GLM index indicates *E. pacifica* and *T. spinifera* were lower than average. Overall, there is coherence between the two time series derived from both modeling approaches (*E. pacifica*, *r* = 0.64, *p* < 0.01; *T. spinifera*, *r* = 0.66, *p* < 0.01), but the limited 2020 sampling likely impacted delta-GLM approach; the SDM approach is independent of the 2020 observations. Spatially, the SDM predicted strong positive anomalies for *T. spinifera* throughout the coast of California and near average conditions for *E. pacifica* (Fig. 4b).

The delta-GLM estimates from the 2020 survey were thus inconsistent with the predictions from the *T. spinifera* SDM, which suggested a return to higher abundance that may be attributed to favorable upwelling conditions during the previous winter^18,28^. Other observations also support that krill abundance in 2020 was likely higher rather than lower. The breeding success of a krill-dependent seabird, the Cassin’s auklet (*Ptychoramphus aleuticus*)^29^ increased in 2020 following a sharp decline in 2019 that coincided with low regional krill abundance (Fig. 4 and Supplementary Figs. 7 and 8), and the 2020 survey coincided with an unusual early occurrence of ~50 blue whales (*Balaenoptera musculus*) aggregated near the Farallon Islands that were feeding on high-density krill swarms^30^. Although the SDM and delta-GLM estimates were largely in accordance prior to 2020, it’s important to note that the SDM by design cannot capture fine-scale patchiness (fixed station data), which likely introduces some uncertainty in predictions (Supplementary Fig. 9). Similarly, the lack of a relationship between relative abundance and SE described previously (Fig. 2) suggests that patchiness (high variability among observations) does not abate with increasing abundance and alternative modeling and model consensus approaches may benefit future efforts^31^.

### Implications for ecosystem monitoring and assessment

Our synthesis provides an optimistic outlook for coping with the loss of ecosystem science data streams needed for fishery assessments. A diversified approach involving application of models and auxiliary observation data streams is promising for monitoring the ecosystem status and maintaining strategic advice for stakeholders during the COVID-19 era. Further, the synthesis and methodology can be easily extended to assess future survey planning when faced with either reduced budgetary constraints or the need to optimize existing survey effort, while maintaining robust estimates of ecosystem conditions. The three-pronged modeling approach involving survey effort simulations, seabird (or other predator) indicators, and species distribution modeling can potentially be extended to any fishery and ecosystem survey that monitors status trends of ecosystem indicators. Notably, we emphasize that our modeling approach works because of the continuous data stream (i.e., no previous cancellations) and should be considered a stopgap measure for a data-poor year and not a replacement for data collection. Furthermore, given the scope of the survey and its use in various stock assessment and ecosystem monitoring frameworks, we did not determine whether or not it truly matters if a survey is missed or if partial data collection may result in increased uncertainty. Future fishery stock assessments should examine this issue and conduct simulations to better understand survey effort reduction or unforeseen cancellations^12^. We maintain that limited survey effort, combined with other ecosystem modeling tools (e.g., from seabirds and SDMs), provides important context for monitoring the health and state of a marine ecosystem during data-poor situations and can be applied to optimize other surveys.

Survey effort simulations provide a powerful tool for assessing strength and uncertainty of ecosystem indicators and can inform future modeling studies and strategic sampling design^12,13^. Signals from seabirds are informative and should be explored to provide important context on connections between fished resources and dependent predators^24^. In our case study, new seabird diet models provided increased confidence of estimates of fish abundance and are powerful, especially during data-poor sampling years. SDMs are helpful for filling information gaps, but often are used to predict species habitat suitability (i.e., probability of occurrence) and not abundance^32^, but they can have limited ecosystem-assessment capabilities if the model performs poorly on novel environmental conditions, such as recent ocean-climate warming events^33^. However, our krill modeling approach was parameterized and trained on surveyed abundance data and historical ocean conditions, thereby providing a novel source of spatio-temporal information for tracking conditions of a critical food-web species.

Planning for potential unavoidable and limited sampling effort should be a priority for all long-term fishery and ecosystem-assessment surveys. The COVID-19 pandemic left a significant mark on marine ecosystem monitoring studies and many ecosystem indicators designed to inform fishery management will likely not be updated, at least without a number of caveats^4^. On the US West Coast, several major surveys were canceled^3^ and the loss of 2020 data may result in increased uncertainty in future stock and ecosystem assessments^4,14^. For example, cancellation of the coastwide groundfish bottom survey, Pacific hake survey, and spring and summer coastal pelagic species surveys leaves a large information gap^3^. The COVID-19 pandemic has also impacted fishery landings due to safety and reduced economic demand^5,34^. In the US West Coast region, fishery landings during March–July 2020 were 31% lower than the previous 5-year period (2015–19 median) and total commercial ex-vessel revenue through October 2020 was reportedly 14% lower^5,35^. Therefore, to better understand these potential fishery economic losses, ecosystem indicators, such as those quantified here (e.g., groundfish, anchovy, and market squid), may benefit fishery assessments by providing strategic advice on marine ecosystem state during the COVID-19 era.

In addition to providing recruitment indicators for a suite of commercially important taxa such as groundfish and market squid^36,37^, our ecosystem survey has played a pivotal role for informing salmon management, understanding unusual marine mammal mortality events, and unraveling impacts from heatwaves to understand and mitigate whale entanglements in fishing gear^27,38^. Thus, losing ecosystem monitoring data may be directly related to loss of insight for informing dynamic ocean management, leading to increased uncertainty^7^. The ecosystem status indicators presented here may be the only information available during 2020, to assess trends and variability of epipelagic forage species in this region. For example, abundance of YOY rockfish, sanddabs, hake, and market squid are below average, whereas the multi-year persistence of high anchovy abundance continues to dominate. Although reduced sampling likely impacted krill assessments, SDM approaches indicate krill species abundance (e.g., *T. spinifera*) should be higher than average, signaling a recovery from the 2019 large marine heatwave^9^. Ecosystem impacts from long-term change and recent unprecedented ocean-climate variability makes it difficult to lose an observation year; because of higher frequency fluctuation of environmental conditions, loss or monitoring data will reduce predictability of ecosystem state^6,27,39^. To better prepare for this uncertainty, evaluation and parameterization of species interactions, coupled with ocean ecosystem models and sampling simulation studies, will contribute to strategic ecosystem-based fishery management.

Updates of many ecosystem indicators will be permanently lost due to the pandemic^4^. Our synthesis provides a basic framework and example for attempting to both recover and validate indicators informed by sparse data, and highlights the need for integration across available data streams, predictive ecosystem modeling, and ultimately a lesson in survey preparedness. Although NOAA fishery-independent surveys were mostly canceled during 2020^3,4^, we were fortunate in our ability to be flexible and respond quickly to develop a contract for chartering a commercial fishing vessel to recover some survey effort. The inspiration from the recent unavoidable survey effort reduction workshop^14^ was invaluable for planning the limited survey and developing a power analysis to evaluate sensitivity and uncertainly of indicators derived from imperfect sampling. We recommend that other fisheries and ecosystem surveys conduct an analysis of the implications of unavoidable survey effort reduction, develop partnerships with researchers investigating top predator population and foraging ecology, and incorporate predictive ecosystem models to their best ability^10^. Seabirds are excellent ecosystem indicators and can contribute substantial information content in the absence and or minimization of fishery and ecosystem survey effort. Further, monitoring of seabirds and marine mammal populations at their colonies occurs in remote field locations via small research teams, making them ideal monitoring tools during a pandemic. In addition, as the charter vessel was unable to deploy oceanographic equipment, we also designed alternative survey plans using robotics, such as oceanographic gliders, equipped with acoustics to monitor fine-scale distribution of krill and forage fish. Although we were ultimately not able to deploy these devices in a time-effective manner, autonomous vehicles may offer substantial opportunities for integrating additional options into existing surveys and could have benefited ecosystem assessments during the COVID-19 era. We urge ecosystem scientists and resource managers to review what existing tools are available to inform ecosystem condition in the absence and or minimization of observational data. Although preparing for the unexpected is difficult, a diversified modeling approach may help overcome fishery management challenges attributed to the pandemic and future reductions of ecosystem monitoring.

## Methods

### Ecosystem-assessment survey and environmental setting

This study uses ecosystem oceanography data derived exclusively from the NOAA RREAS, stored on NOAA-ERDDAP and reported by the California Current Integrated Ecosystem Assessment in an annual Ecosystem Status Report^9^ provided to the Pacific Fisheries Management Council (PFMC). Since 1983, the RREAS generally operates from early May through mid-June to assess ocean conditions and the abundance and distribution of YOY rockfish and other YOY groundfish (such as Pacific hake and sanddabs), in order to inform fisheries stock assessment models and ecosystem status reports. For stock assessments, abundance estimates of pelagic YOY are developed from survey data and are used as an index of year class strength for a number of commercially and recreationally important groundfish in the West Coast fishery (see PFMC in “Data Availability”). As groundfish species experience considerable variability in cohort strength from year to year, with the difference often spanning several orders of magnitude^40^ and typically being weakly related to spawner abundance^41^, the intent of the survey indices is to improve model forecasts of the abundance and availability of these year classes to commercial and recreational fisheries^36,42,43^. In doing so, continuous time series are of the greatest utility, as the objective is to ensure that strong year classes that might substantially alter population trajectories are recognized prior to entering the fisheries. Since 1990, the survey has also quantified other epipelagic micronekton, with an emphasis on ecologically important forage species, to support a growing array of ecosystem studies and to provide ecosystem indicators to marine resource managers^16,22^.

From 1983 to 2003, the survey operated solely off of central California (between ~36° N and 38° N latitude), the core survey area off California, with mid-water trawling occurring on the continental shelf (<200 m), outer slope (>200–1500 m), and in deeper waters (>1500 m) (Fig. 1 and Supplementary Figs. 1–3). Since 2004, the survey has covered most of the California coastline, from the US/Mexico maritime border to the California/Oregon border^22^ (Fig. 1). Mid-water trawls were collected at fixed sampling stations during the night using a modified Cobb mid-water trawl with a 9.5 mm cod-end liner; 15 min tows were made at each station with a headrope depth of 30 m, except at stations where shallow bottom depths (<55 m) where headrope depth was 10, in order to avoid bottom contact. After each trawl, all taxa were enumerated and relative species abundance was measured as CPUE per trawl. For a synthesis of the spatial distribution and temporal variability of micronekton and their ecosystem considerations, see refs. ^17,22^. Briefly, species distribution patterns reflect onshore oceanographic gradients and reflect recruitment patterns related to winter and spring ocean conditions^16,26^, and are linked to productivity, distribution and feeding ecology of seabird, marine mammals, and commercially harvested species such as salmon^27,38^. During the past decade, ocean-climate conditions in the CCLME have been highly anomalous^44^ with impacts ranging from persistent (multi-year) marine heatwaves, harmful algal blooms, and marked fluctuations of biodiversity and recruitment of pelagic fish species^16,23^.

### Pandemic response and sampling procedure

In response to the COVID-19 pandemic, the usual NOAA research ship platform was not available for the 2020 survey^3^. Consequently, the NOAA Cooperative Research Program provided support for a contracted commercial charter to conduct mid-water trawling to ensure an uninterrupted 38-year time series within the core study area. No data were collected from the broader study region in 2020 and those regional time series are not updatable at this time. Additional critical observational data such as oceanographic data (including profiles of the water column and environmental DNA sampling), hydroacoustics to map krill and fish distribution, and visual surveys to map seabird and mammal distribution and abundance were not able to be collected. Survey team scientists participated in training the vessel crew for two nights on how to fish the gear and sample catches. Subsequently, catches (or subsamples for very large trawls) were transported to the laboratory every 1–2 days for taxonomic identification, enumeration, length measurements, and sample archiving. Temperature-depth recorders were attached to the trawl headrope and footrope, and data downloaded every 1–2 days, to ensure that trawl protocols (target headrope depth and duration of trawl) were followed. Through a combination of bad weather and gear damage, only 15 trawls were conducted in 2020 in the historical core area compared to an average of 62 trawls per year between 2004 and 2019 (with an average of 140 for the entire survey area; Fig. 1). In addition, survey effort did not begin until 8 June and extended through 25 June, later in the season than typical survey effort (May–June), and after the period of peak abundance for pelagic juvenile rockfish, which have a strongly seasonal abundance cycle^36^. The smaller size of the chartered fishing vessel, combined with poor weather conditions over the duration of the survey, also resulted in unbalanced sampling, in which more samples were collected in waters over the continental shelf than over the outer slope (Fig. 1 and Supplementary Figs. 1–4).

### Seabird observations

Seabird diet composition was determined through observations of prey delivered to dependent offspring at Southeast Farallon Island (1983–2020) and subsequent conversion to mass using length–weight regressions^17^. For common murre, which provision chicks by carrying single prey items lengthwise in their bill, prey was visually identified using binoculars during standardized daily feeding watches throughout the peak chick rearing period, late May to early July. During observations, all prey items were identified to the lowest possible taxon based on color, body shape, tail shape, and shape and position of fins. Species-group categories were used, i.e., juvenile rockfishes, northern anchovy/Pacific sardine (*Sardinops sagax*), smelt (Osmeridae spp.), market squid, salmon (*Onchoronchys* spp.), flatfishes (Pleuronectidae) including sanddabs, sandlance (*Ammodytes hexapterus*), lingcod (*Ophiodon elongatus*), sculpins (Cottidae), Pacific saury (*Cololabis saira*), and unidentified prey.

### Statistical analysis

Using our knowledge of ecosystem dynamics, we applied four analytical tools to generate and evaluate the robustness of ecosystem indicators for 2020 given unavoidable survey effort reduction due to the COVID-19 pandemic. We (1) updated ecosystem indicators to better account for spatially biased sampling; (2) estimated changes in accuracy and precision of ecosystem indicators due to lost survey effort; (3) predicted the 2020 indicators based on the relationship between past indicators and an independent data source (seabird diet) that was collected in 2020; and (4) predicted the distribution and abundance of krill species (key components of the marine food web) using a spatially explicit model based on past data. These data are, at present, the only NOAA fishery-independent observations available to assess ecosystem condition and status for this region and time period.

### Ecosystem indicators

The primary objective of the RREAS is to derive standardized estimates of species CPUE (relative abundance indices) to inform future stock and ecosystem assessments, and process studies^23,36^. Here we estimate and update indicator time series for YOY rockfish, YOY sanddabs, YOY Pacific hake, adult northern anchovy, market squid, myctophids, octopus, and krill in the core area for the years 1990–2020 (Fig. 1). Given the sampling challenges in 2020 and the fact that most survey effort occurred in nearshore waters, abundance indices were estimated using a delta-GLM approach (also referred to as a hurdle model), in which data were fitted separately to a binomial GLM and a lognormal positive model that used only data with positive catches. The fixed-year coefficients (back transformed to proportion positive catch and abundance given positive catch) are multiplied to produce the annual index. This approach can account for unbalanced sampling through the inclusion of spatial and temporal covariates in both the binomial and positive model^45,46^. The delta-GLM method is routinely used to generate relative abundance indices from fisheries survey and catch rate data to inform stock assessments^19^, and as such is also the standard approach used to develop YOY recruitment indices from this survey for use in stock assessments^36,42,43^. As spatial covariates, we considered the effect of station, region, depth, and the interaction between region and depth, selecting the best set for each model for each species using Akaike’s Information Criteria. For YOY rockfish, we also considered a temporal covariate (Julian day bin) to account for known seasonal variability in the availability of YOY rockfish. Uncertainty in the year effects was quantified by running the model in a Bayesian framework with vague priors and computing 95% credible intervals using the package “rstanarm”^47^ in R version 3.6.3^48^ within RStudio (version 3.5.3). The resulting indices and uncertainty estimates were log(*x* + 1)-transformed and *z*-scores were produced from the log-transformed data, consistent with previous reporting of these ecosystem indicators as anomalies around their long-term average abundance levels^9^.

### Survey effort reduction simulations

To verify that the abundance indices generated for 2020, after accounting for covariates, were not systematically biased, we simulated comparable effort reduction in past years. For each year from 1990 to 2019, we removed all trawls from the focal year, except for the last trawl taken at each of the 15 stations that were sampled in 2020, retaining all data from all other years, and computed the abundance index for the focal year using the same delta-GLM procedure. We then computed the relative bias in the index for each year for each taxon (deviation of the point estimate with reduced sampling relative to the point estimate with all available trawls). In addition, to better understand the trade-off more generally between sample size (number of trawls conducted in a given year) and the uncertainty (standard error) associated with the resulting indices, we evaluated the relationship between SE, sample size-corrected SD, and the index mean across years for each taxon, as well as the relationship between sample size and SE for each taxon within each year. Index SEs and means (in log space, excluding zeros and not adding 1) were taken from the Bayesian delta-GLM using all data (SE increased with the mean in arithmetic space, as built into the lognormal model). Extrapolation of observed SEs to other sample sizes was done using the standard scaling relationship, ${{{{{\rm{SE}}}}}}={{{{{\rm{SD}}}}}}/\sqrt{n}$, where *n* is the total number of trawls conducted in that year. We confirmed that this approach is reasonable for most taxon and year combinations by randomly excluding all but *n* trawls from a past focal year, recomputing the SE for a range of different *n* and several different focal years, and comparing the results to those predicted by the standard scaling relationship. Purely random exclusion of trawls and more realistic random exclusion of trawls (e.g., consecutive trawls from randomly selected nights up to *n* trawls) gave similar results.

### Seabird diet and indicator model

Seabird diet varies with the (relative) abundance of their prey species in the surrounding marine ecosystem^17,20,38^. Previous studies used bivariate linear regression to examine the relationship between seabird diet (mean proportion of prey fed to common murre chicks on Farallon Island) and abundance indices of forage species (specifically YOY rockfish) derived from the RREAS for the years 1983 to 2001^21^. Given the paucity of RREAS data in 2020, but the continued collection of common murre diet data, we used this relationship and the 2020 murre diet data to predict 2020 RREAS indices. We first updated the model^21^ to include data from 1983 to 2019 (18 additional years) and used survey abundance indices derived from the new delta-GLM approach. We specifically modeled the relationship between the proportion of YOY rockfish in the common murre diet and the abundance index for YOY rockfish standardized to age 100 days^36^. As common murre consume primarily YOY rockfish or anchovy^17^ (Supplementary Fig. S.7), we also modeled the relationship between the proportion of anchovy in the common murre diet and the abundance index for anchovy for the years 1990–2019 (earlier years excluded due to lack of standardization of anchovy counts). We then used these regression models to predict YOY rockfish and anchovy abundance indices from 2020 common murre diet observations and compared these to the 2020 abundance indices generated from the survey itself. This comparison allowed us to assess confidence and uncertainty in these estimates. We note that total YOY rockfish represent a collection of 10+ species, which covary highly^17,36^, whereas anchovy is just one species, with both taxa having episodic periods of elevated abundance that persist for several years^16,23^.

### Krill species distribution model

Krill distribution is patchy and the mid-water trawl samples the relative abundance of krill at the scale of swarms^26^. Aggregations of krill swarms are concentrated at 30 m, the approximate average mixed-layer depth in the study area, and may range for several km in the alongshore direction^27^. Further, the spatial mean of krill CPUE and coefficient of variation (CV) are measures of relative abundance and patchiness, respectively (Supplementary Fig. 8). SDMs have been previously created to understand drivers of the distribution and relative abundance (CPUE) of the two dominant krill species in the CCE, *T. spinifera* and *E. pacifica*^18^. Briefly, using krill CPUE data derived from the RREAS trawls from 2002 to 2018, a boosted regression tree approach was used to integrate species-specific abundances with environmental data. The final models for each species included different combination of winter preconditioning upwelling dynamics from remote-sensing and a data-assimilative Regional Oceanographic Modeling System^49,50^, static geomorphic features (e.g., depth and distance from shore), and spring mesoscale oceanographic conditions that reflect the ocean state during the survey period. The models reproduced the neritic distribution of *T. spinifera* and outer slope association of *E. pacifica*, accurately predicted species responses to climate events (e.g., 2014-16 heatwave and ENSO), and were independently evaluated with krill predator (seabird and mammal) distributions that showed predators were present in regions with predicted high krill abundance^18^.

Using this developed model and environmental data from 2019 and 2020, we predicted the distribution and abundance of krill during these 2 years to evaluate the ability of the model, to perform on data outside of the training data set and provide more information on ecosystem state given reduced sampling in 2020. The model predictions for 2019 and 2020 were compared to observations (Supplementary Fig. 9) and model-based estimates (i.e., delta-GLM) of krill from RREAS mid-water trawls. Therefore, our approach for krill species involved a consensus of two modeling techniques. We created a mean krill CPUE time series from the SDM based on predictions at all the core stations to make an appropriate comparison to the delta-GLM index. Further, for the SDM, we mapped krill species abundance anomalies during each year relative to the long-term mean of predictions from 2002 to 2018 and, therefore, the anomalies are directly comparable to those in ref. ^18^. Finally, we compared the observed CV among stations in the core region to model predictions by subtracting the observed CPUE from the predicted CPUE to see how krill patchiness related to model results.

### Reporting summary

Further information on research design is available in the Nature Research Reporting Summary linked to this article.

## Supplementary information

Supplementary Information File

Description of Additional Supplementary Files

Supplementary Software

Reporting Summary

## Data Availability

The data generated in this study are provided in the Supplementary Information. All data pertaining to ecosystem indicators are available from the California Current Integrated Ecosystem Assessment: https://www.integratedecosystemassessment.noaa.gov/regions/california-current-region/index.html. All data from the RREAS is maintained on the NOAA-ERDDAP portal and are freely accessible: NOAA Environmental Research Division Data Acquisition Portal (ERDDAP): https://coastwatch.pfeg.noaa.gov/erddap/tabledap/FED_Rockfish_Catch.html. Pacific Fisheries Management Council (PFMC) https://www.pcouncil.org/stock-assessments-star-reports-stat-reports-rebuilding-analyses-terms-of-reference/. Supplementary Software: Computer code, including model fitting, effort reduction simulation, and application of krill species distribution model, are provided within the Supplementary Software file. Source data are provided with this paper.

## Source data

Source Data
