# Supplementary material for: Diverse integrated ecosystem approach overcomes pandemic-related fisheries monitoring challenges
Source: Nat Commun. 2021 Nov 11;12:6492. doi: 10.1038/s41467-021-26484-5 (PMC8585921; doi:10.1038/s41467-021-26484-5)
Supplement: Supplementary file 1 — Supplementary Information File [file 41467_2021_26484_MOESM1_ESM.pdf]

Supplementary Information File

Diverse integrated ecosystem approach overcomes pandemic-related fisheries monitoring challenges

Jarrod A. Santora[1, 2]* Tanya L. Rogers[1], Megan A. Cimino[3, 4], Keith M. Sakuma[1], Keith D. Hanson[1], E.J. Dick[1], Jaime Jahncke[5], Pete Warzybok[5], John C. Field[1]

[1]Fisheries Ecology Division, Southwest Fisheries Science Center, National Marine Fisheries Service, National Oceanic and Atmospheric Administration, Santa Cruz, California, 95060

[2]Department of Applied Math, University of California Santa Cruz, California, 95060

[3]Institute of Marine Sciences, University of California Santa Cruz, California, 95060

[4]Environmental Research Division, Southwest Fisheries Science Center, National Marine Fisheries Service, National Oceanic and Atmospheric Administration, Monterey, California, 93940

[5]Point Blue Conservation Science, Petaluma, California, 94954

*Corresponding author email: Jarrod.Santora@noaa.gov

Supplementary Figure 1

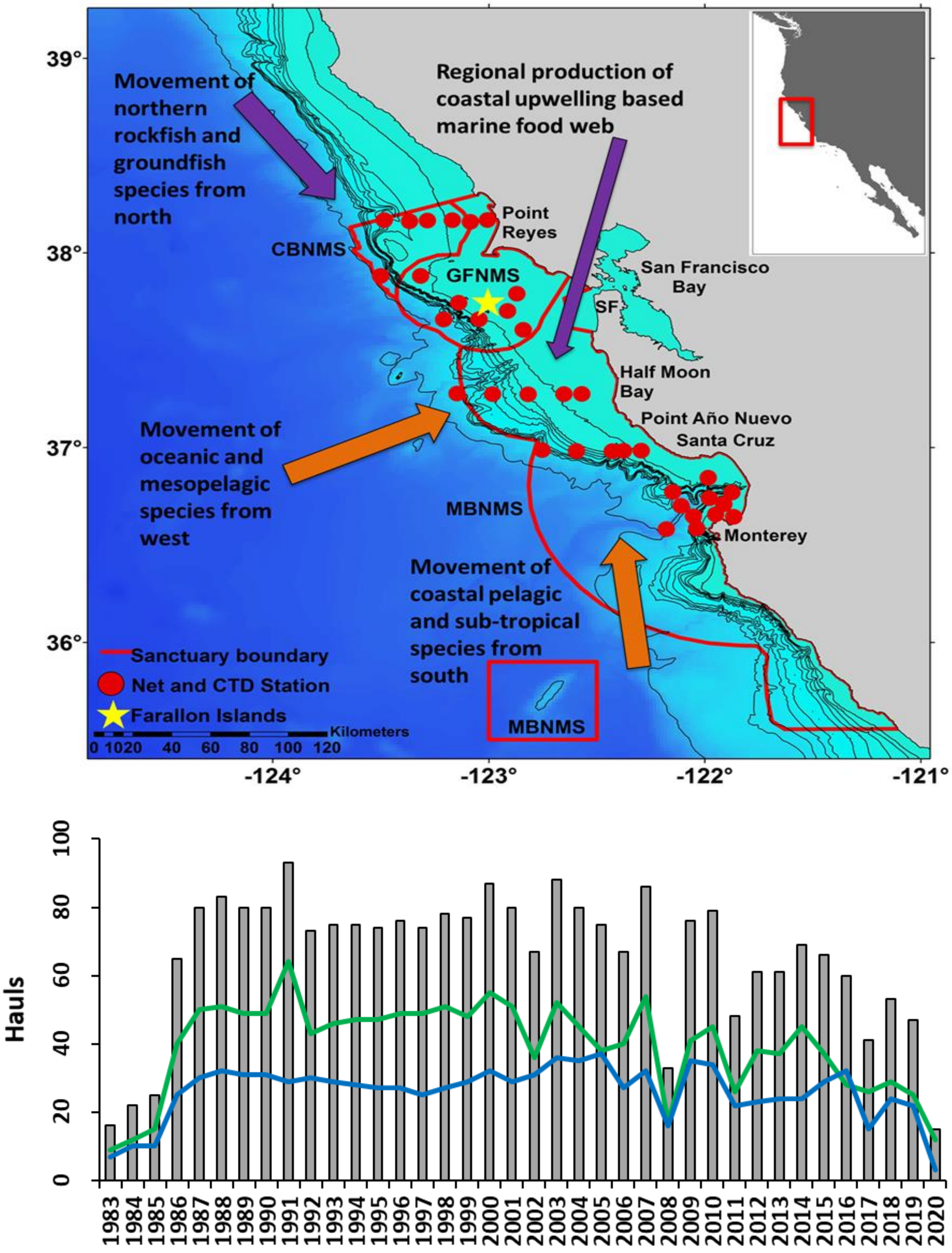

Supplementary Figure 1: (**top**) Long-term 'core' study region of the Rockfish Recruitment and Ecosystem Assessment (RREAS); arrows indicate occurrence and movement of micronekton taxa and biodiversity as summarized per Santora et al. 2017. National Marine Sanctuary (NMS) boundaries are in red, where, CBNMS is Cordell Bank, GFNMS is Gulf of the Farallones, and MBNMS is Monterey Bay. (**bottom**) Summary of the number of mid-water hauls collected in the core region; green line is the number of hauls sampled on the shelf (<200 m) and blue line is the number of hauls collected in deeper outer slope (>200 m). Map created by J. Santora.

Supplementary Figure 2

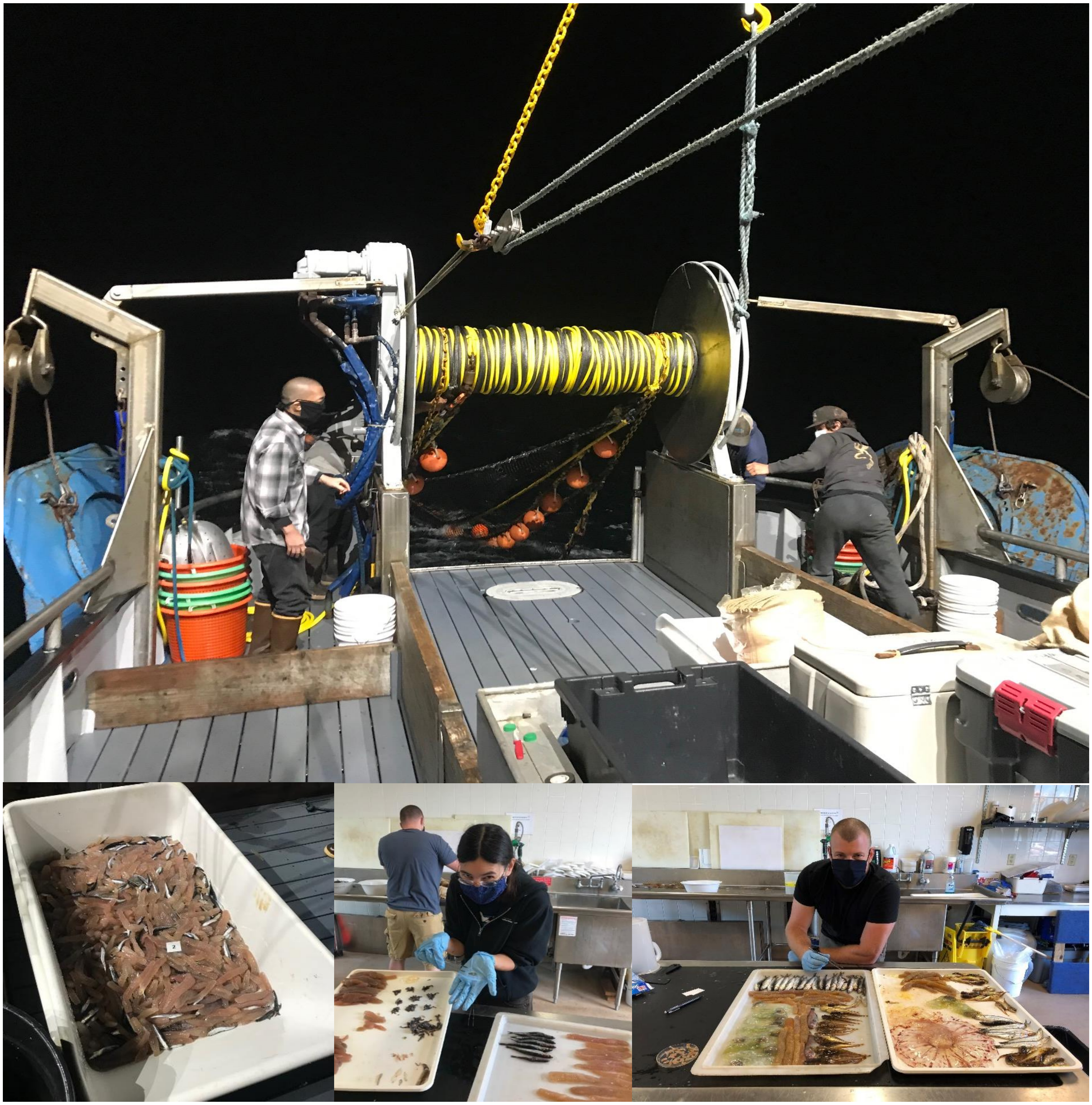

Supplementary Figure 2: (top) Conducting the RREAS mid-water trawl aboard a chartered commercial fishing vessel and training crew to collect and sort the catch. (bottom left) large catch of pyrosomes and northern anchovy. (bottom right) sorting and enumeration of micronekton samples in the NOAA laboratory, all following social distancing and COVID-19 protocols. Photos by K. Sakuma and J.C. Field

Supplementary Figure 3

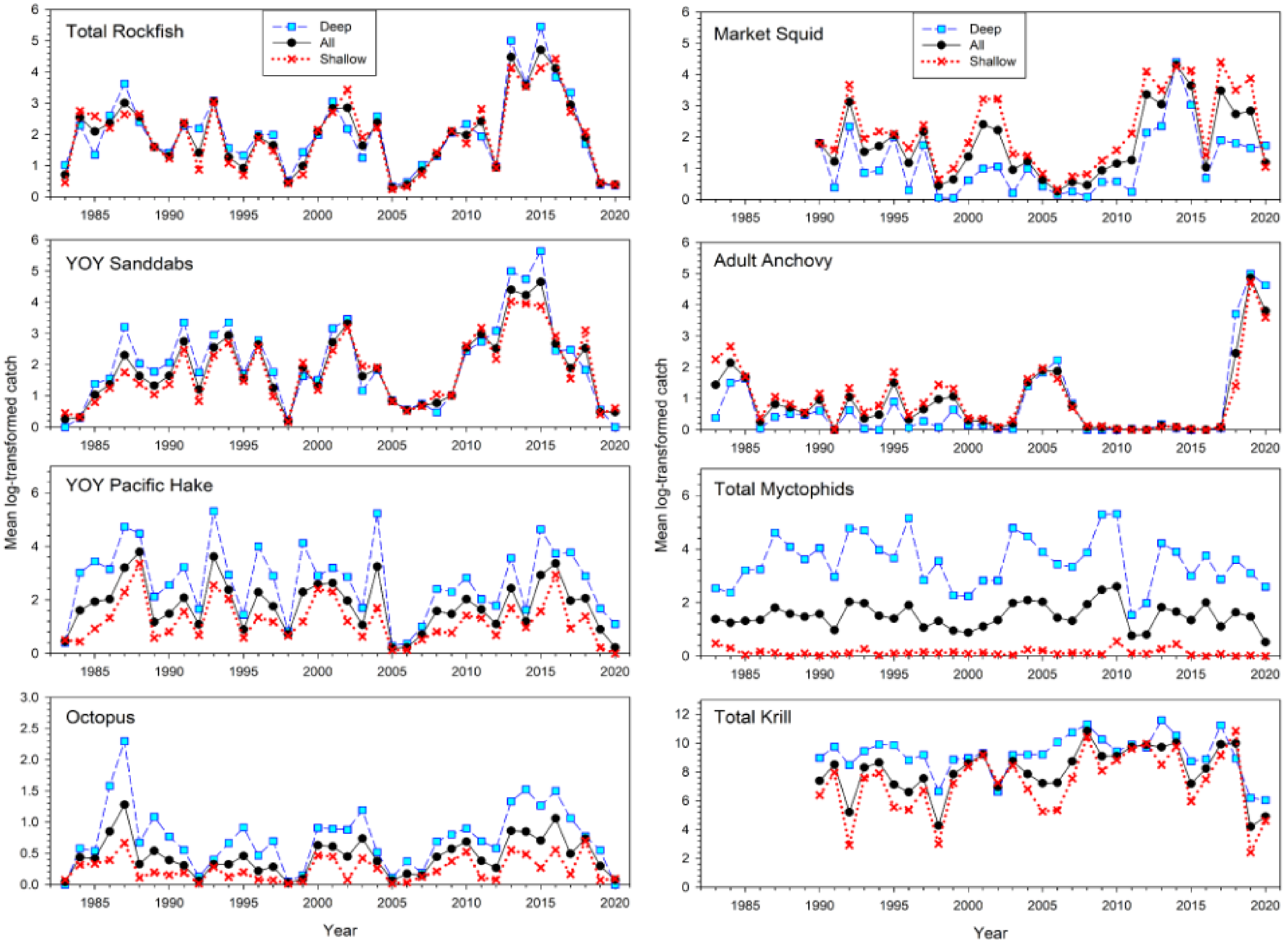

Supplementary Figure 3: Comparison of taxa indices (Mean log-transformed catch) within the RREAS core region over all, deep and shallow sampling locations. Taxa with shallow or offshore habitat affinities may be biased from limited sampling.

Supplementary Figure 4

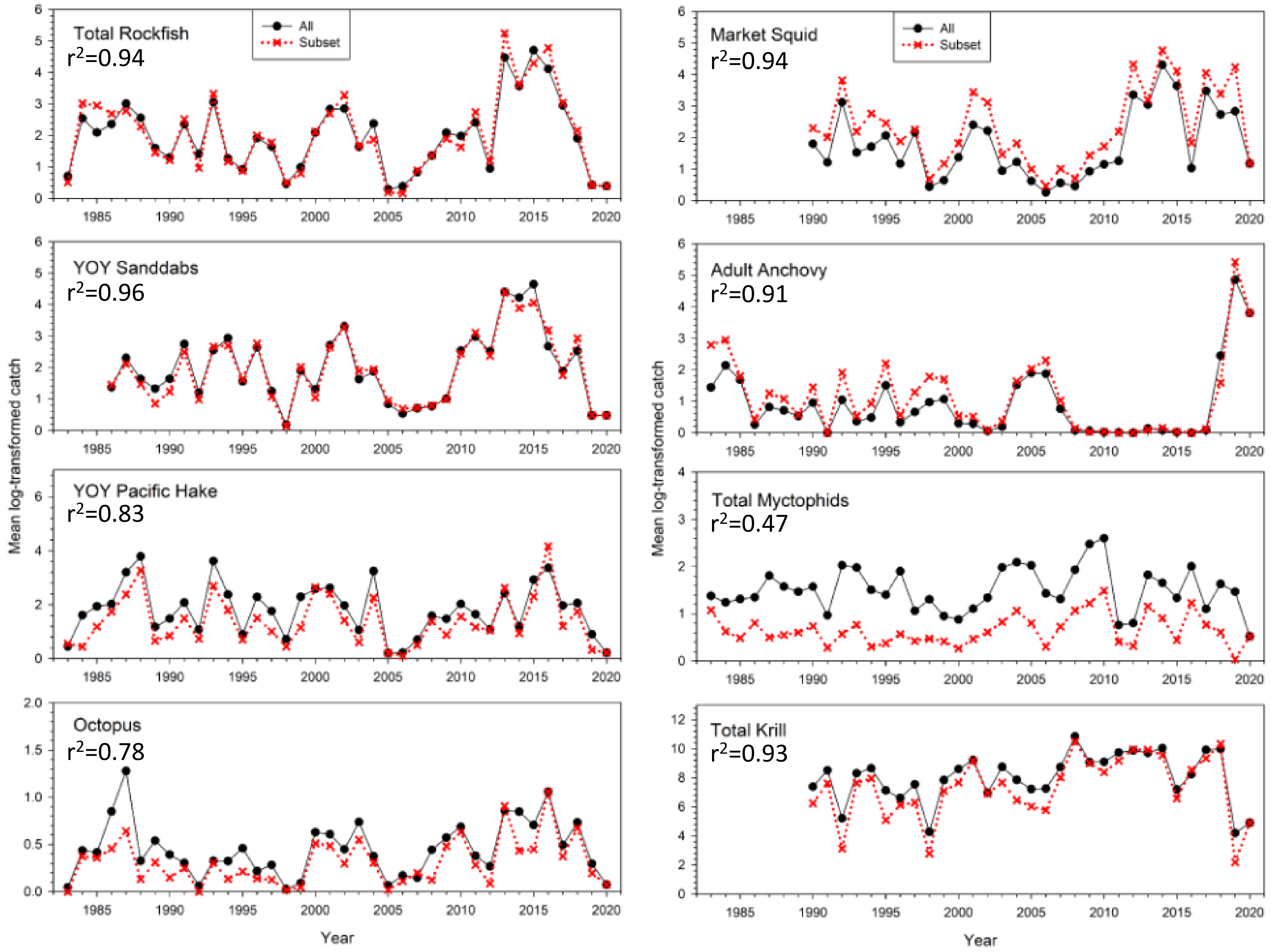

Supplementary Figure 4: Comparison of taxa indices (Mean log-transformed catch) within the RREAS core region for the subset (red) of 15 stations sampled in 2020 vs. all stations (black); r^2 values indicate coherence between series.

Supplementary Figure 5

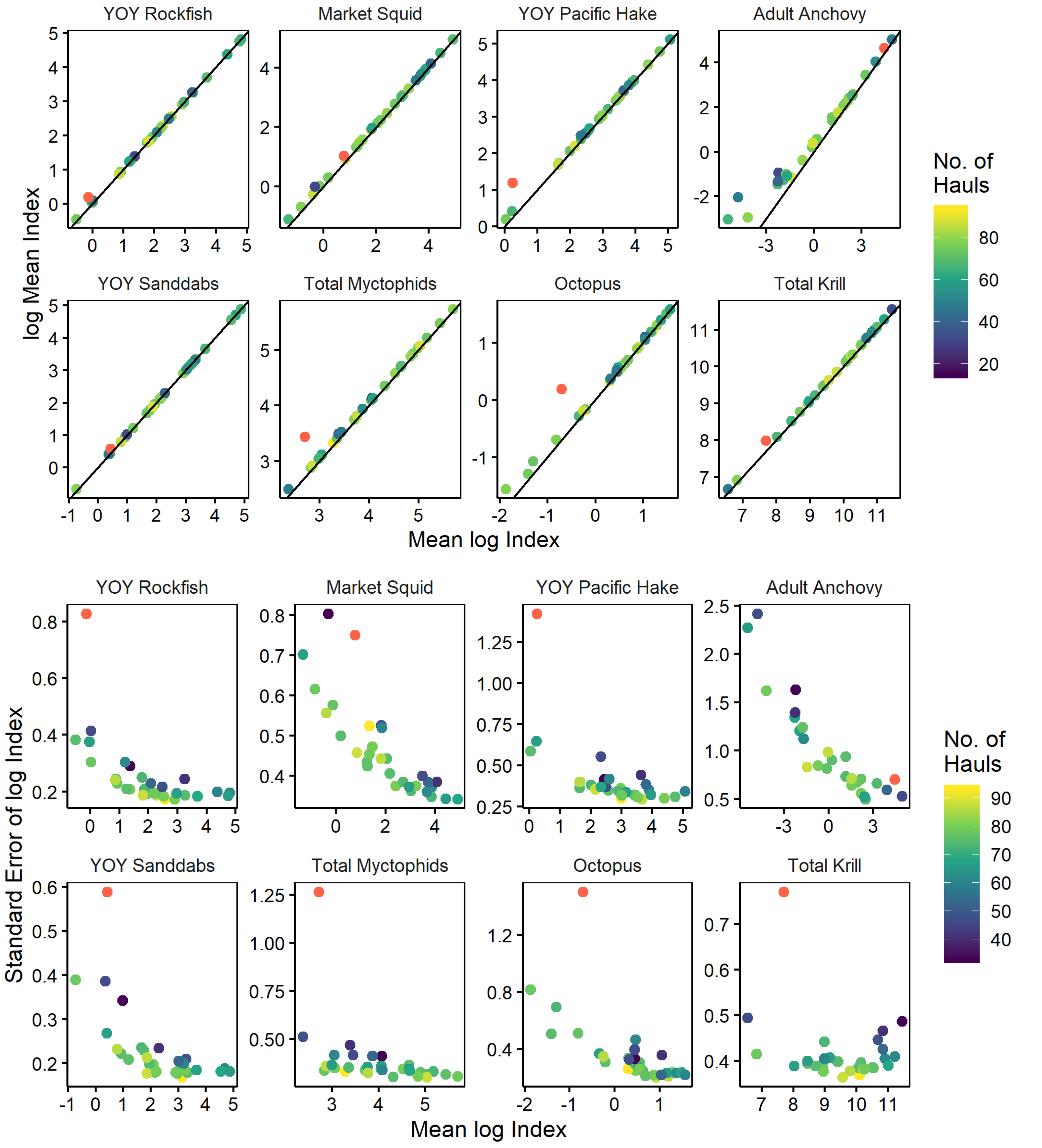

Supplementary Figure 5: Results from STAN delta-GLM model using all data. Each point is a year. Red point is 2020. **(top)** relationship between mean log index and log mean index. Deviations from the 1:1 line reflect variance. (**bottom**) Mean vs. standard error of index on log scale. Effect of sample size is apparent.

Supplementary Figure 6

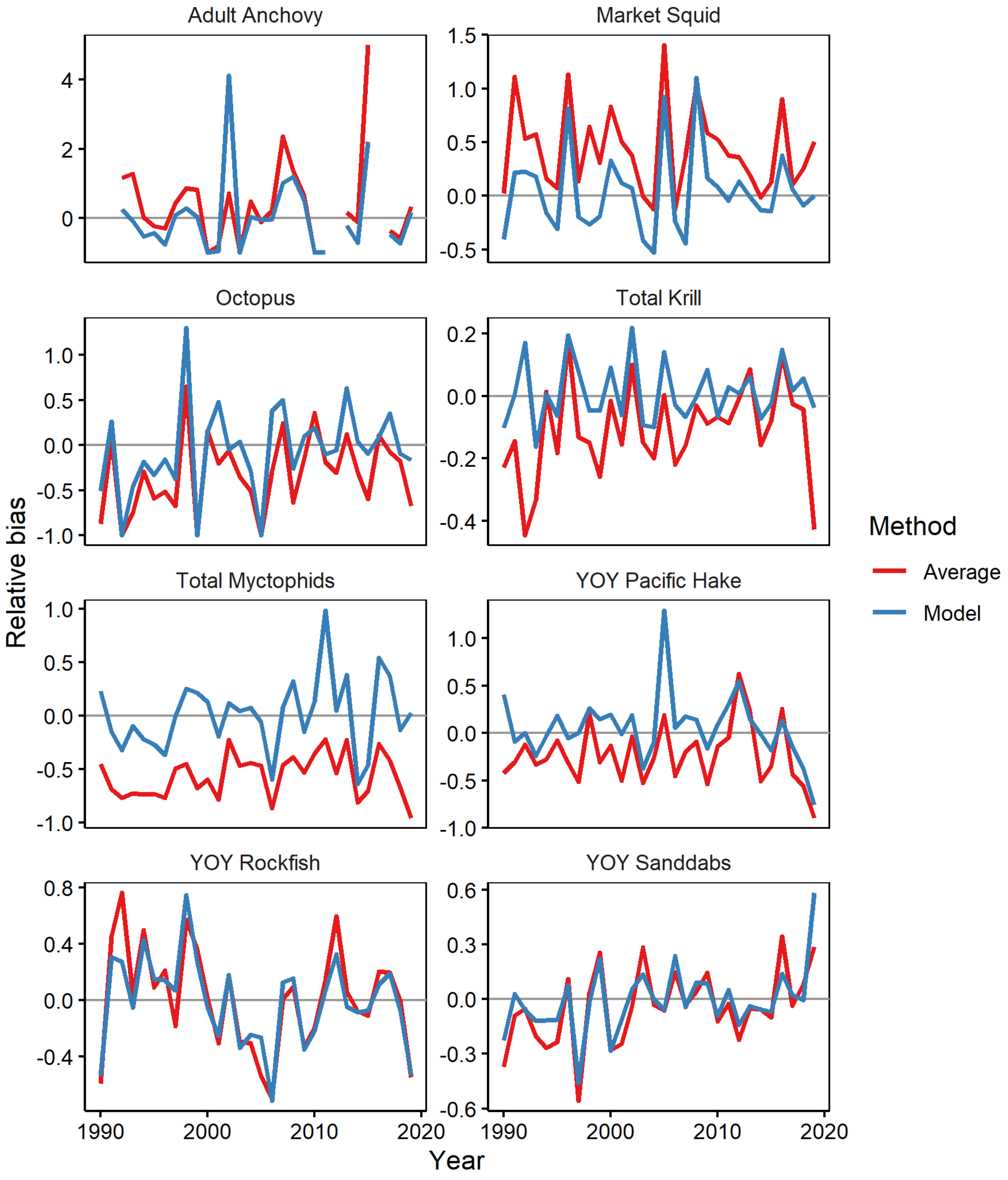

Supplementary Figure 6: Relative bias in the index point estimate using 15 hauls from the 2020 stations vs. all hauls from all stations sampled in a given year, computed as $(x_{2020} - x_{all}) / x_{all}$, for each year 1990-2019. The index was computed either by averaging values of log(CPUE+1) from all available hauls in a given year (red lines) or from the maximum likelihood estimate of a delta-GLM model with spatial covariates (blue lines), as log(MLE+1). For the model-based index, the x_{2020} estimate excludes hauls from the focal year but includes complete data from all other years.

Supplementary Figure 7

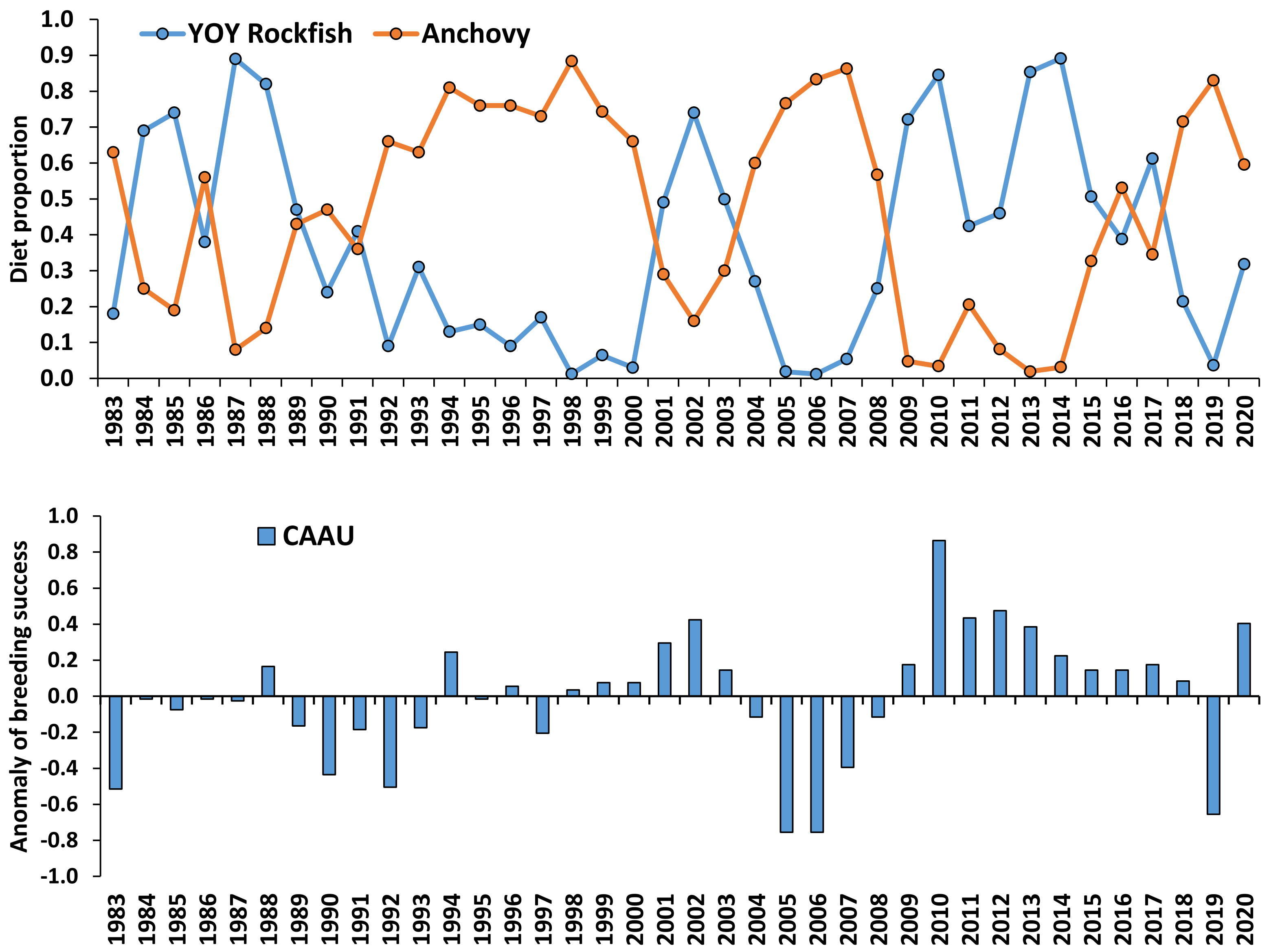

Supplementary Figure 7: (**top**) Diet studies of common murre collected from visual surveys on Southeast Farallon Island (see Fig. S.1); indices are mean proportion of prey (YOY Rockfish and anchovy) fed to chicks and are used to develop regression models between seabird diet and catch indices from the RREAS mid-water trawl survey. (**bottom**) Standardized anomaly of seabird reproductive success (mean chicks fledged per pair) for CAAU, Cassin's auklet (a krill specialist).

Supplementary Figure 8

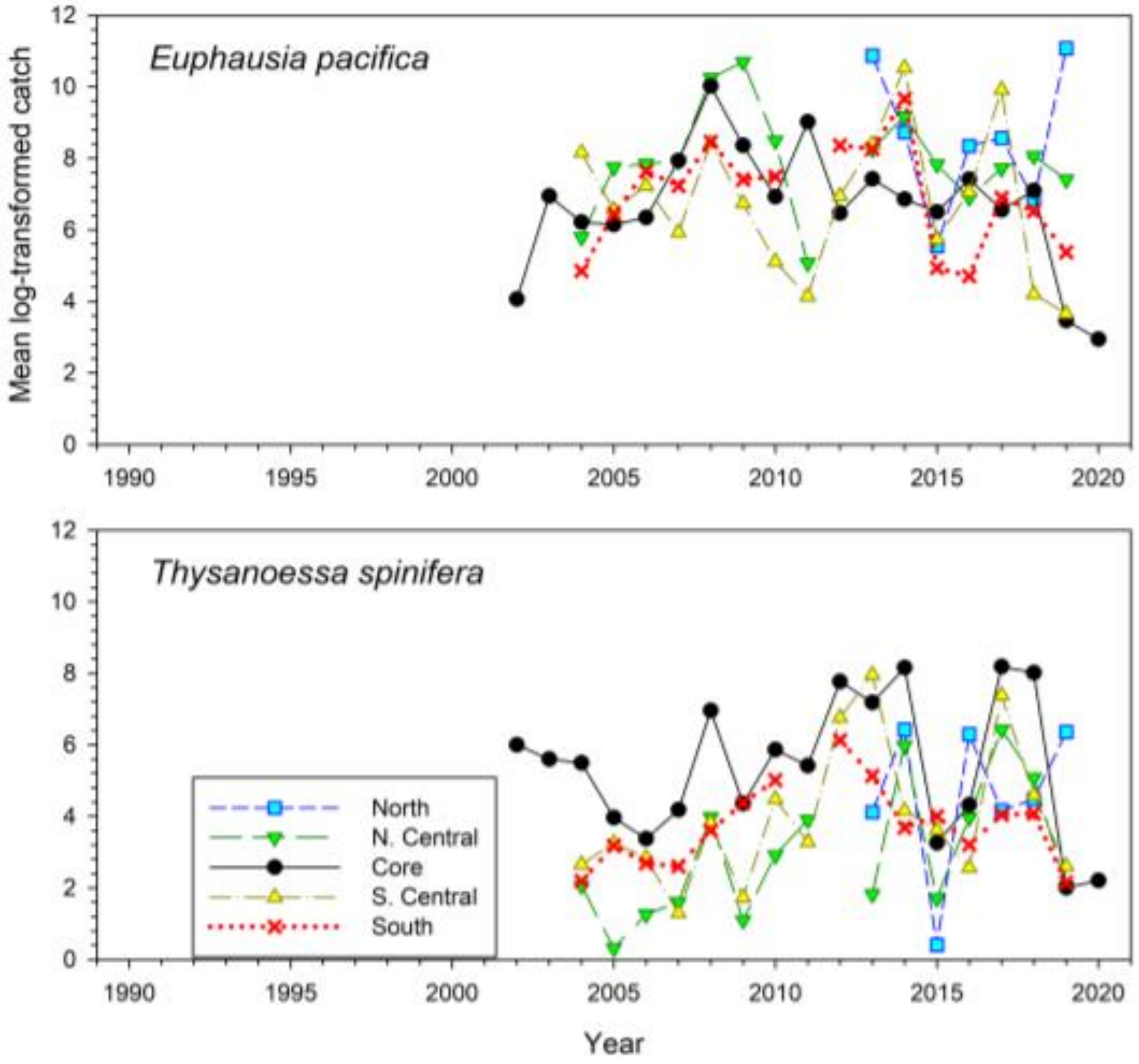

Supplementary Figure 8: Regional indices of krill species CPUE (mean log-transformed catch) derived from the RREAS. (top) *Euphausia pacifica* and (bottom) *Thysanoessa spinifera*. Note estimates from limited sampling in 2020 are problematic for estimating relative abundance patterns. See Figure 1 for extended sampling regions.

Supplementary Figure 9

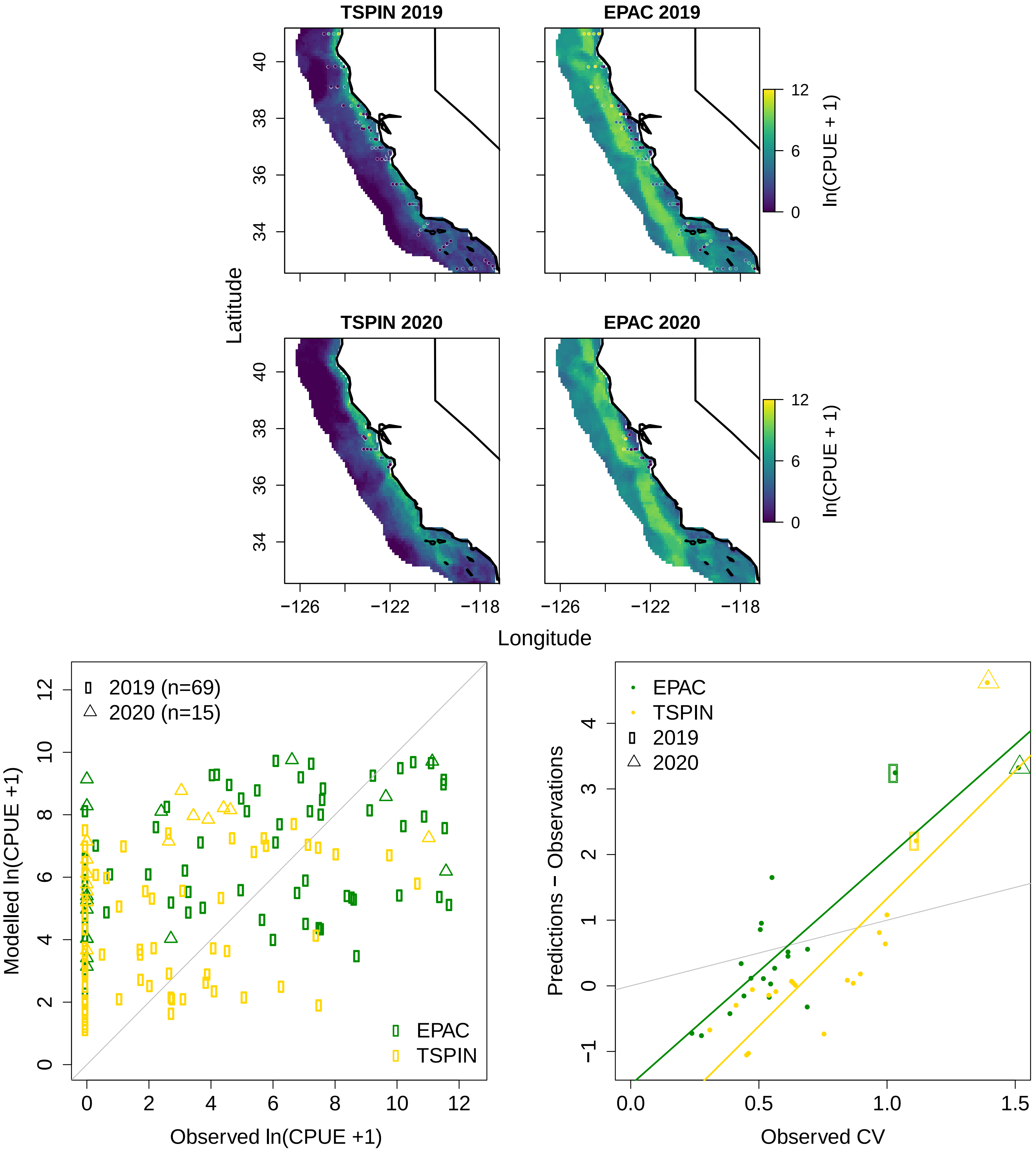

Supplementary Figure 9: Krill species distribution model; EPAC is *Euphausia pacifica* and TSPIN is *Thysanoessa spinifera*. (**top**) comparison between modeled and observed CPUE for 2019-2020; station observations are plotted over predicted surfaces. (**bottom left**) predicted mean CPUE during May 2019 and 2020. (**bottom right**) relationship between differenced CPUE (predicted subtracted from observed values) and observed coefficient of variation (CV), a measure of heterogeneity not incorporated into the krill species distribution model. CPUE is 'catch-per-unit-effort'.
