# Supplementary figures and images for: Diverse integrated ecosystem approach overcomes pandemic-related fisheries monitoring challenges
Source: Nat Commun. 2021 Nov 11;12:6492. doi: 10.1038/s41467-021-26484-5 (PMC8585921; doi:10.1038/s41467-021-26484-5)

**TSPIN 2019**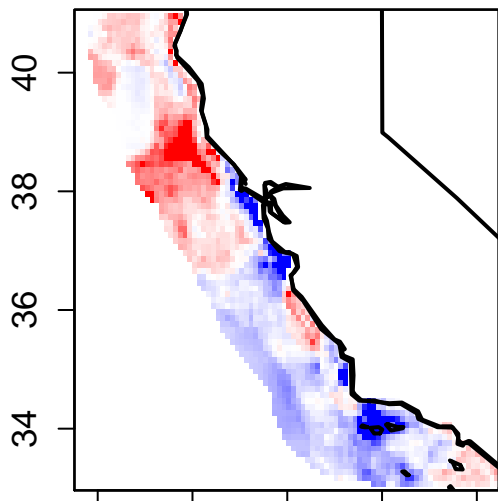**EPAC 2019**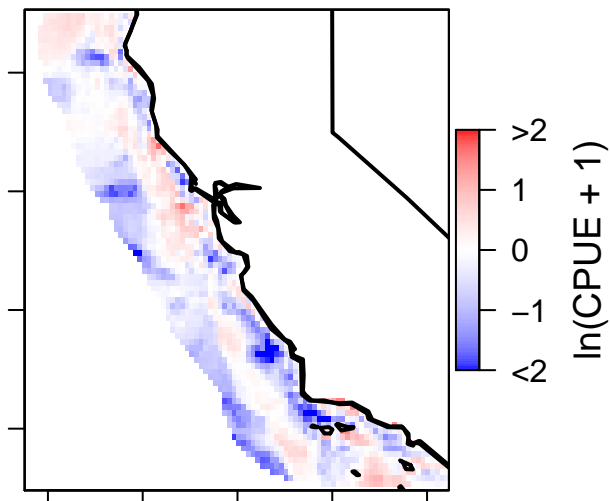**TSPIN 2020**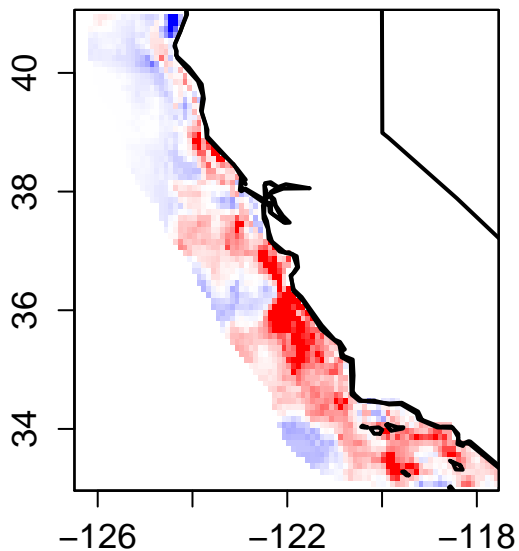**EPAC 2020**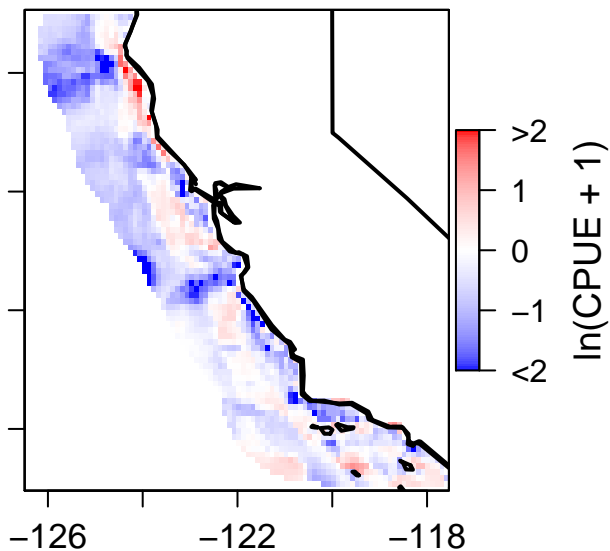

Latitude

Longitude

Supplement: Supplementary file 3 — Supplementary Software [file 41467_2021_26484_MOESM3_ESM.zip › Krill_SDM/figures/EPAC_TSPIN_anomaly_maps.pdf]

**TSPIN 2019**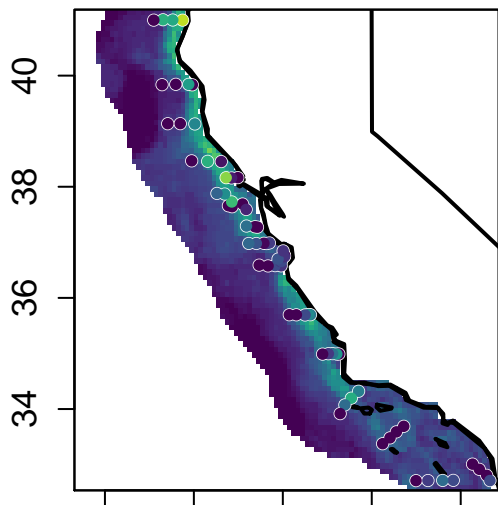**EPAC 2019**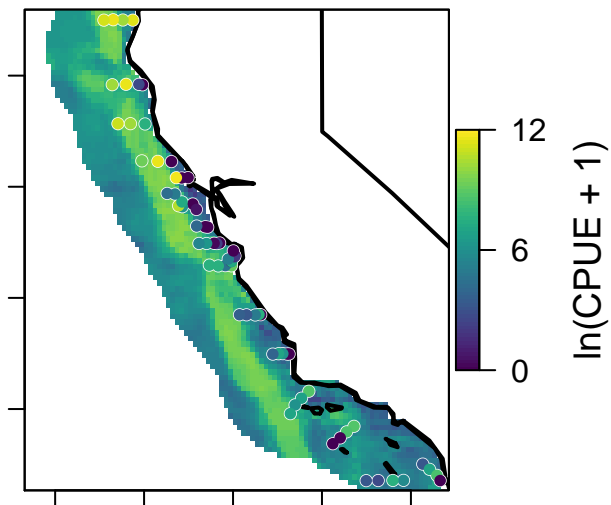**TSPIN 2020**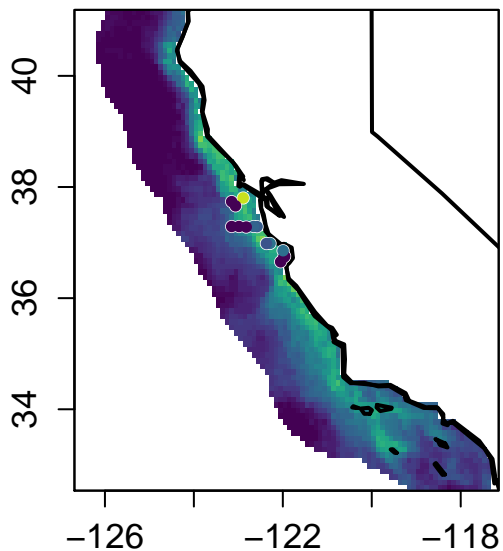**EPAC 2020**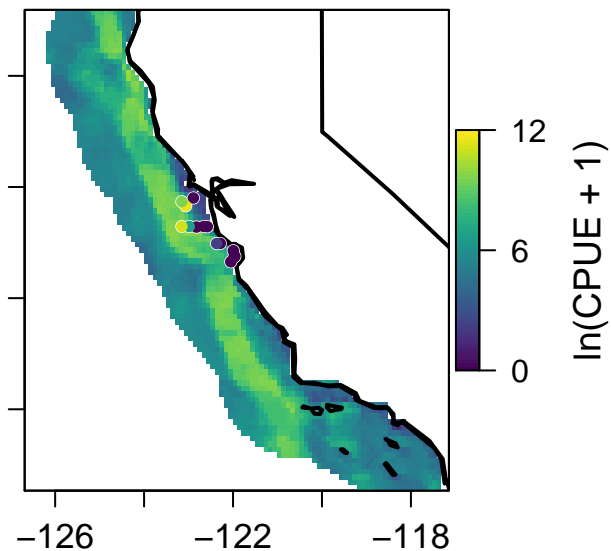

Latitude

Longitude

Supplement: Supplementary file 3 — Supplementary Software [file 41467_2021_26484_MOESM3_ESM.zip › Krill_SDM/figures/EPAC_TSPIN_SDM_mapped.pdf]

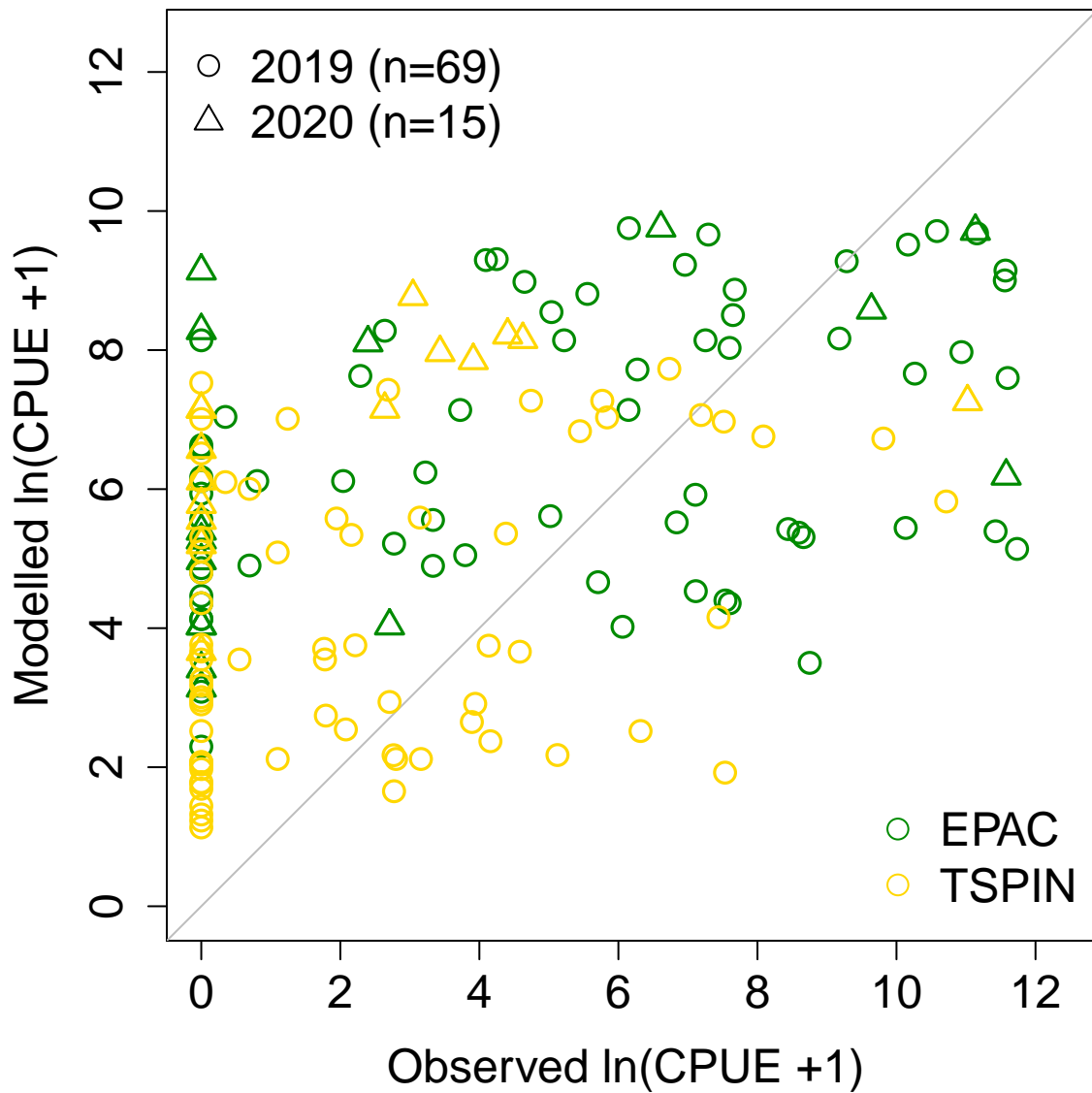

Supplement: Supplementary file 3 — Supplementary Software [file 41467_2021_26484_MOESM3_ESM.zip › Krill_SDM/figures/Model_vs_obs_19_20.pdf]

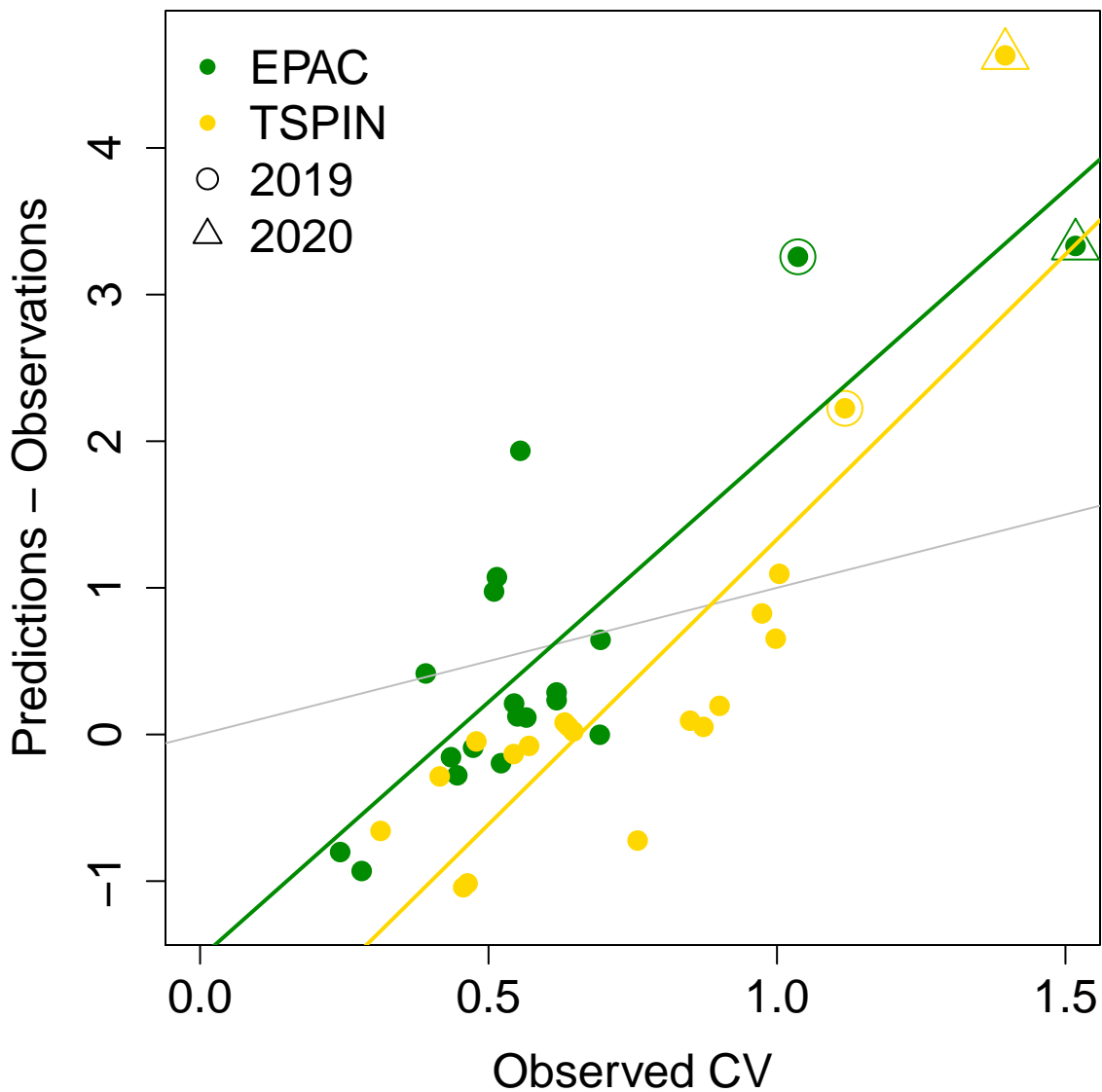

Supplement: Supplementary file 3 — Supplementary Software [file 41467_2021_26484_MOESM3_ESM.zip › Krill_SDM/figures/ObservedCV_vs_predict.obs.pdf]

# EPAC

Standardized Index or CPUE

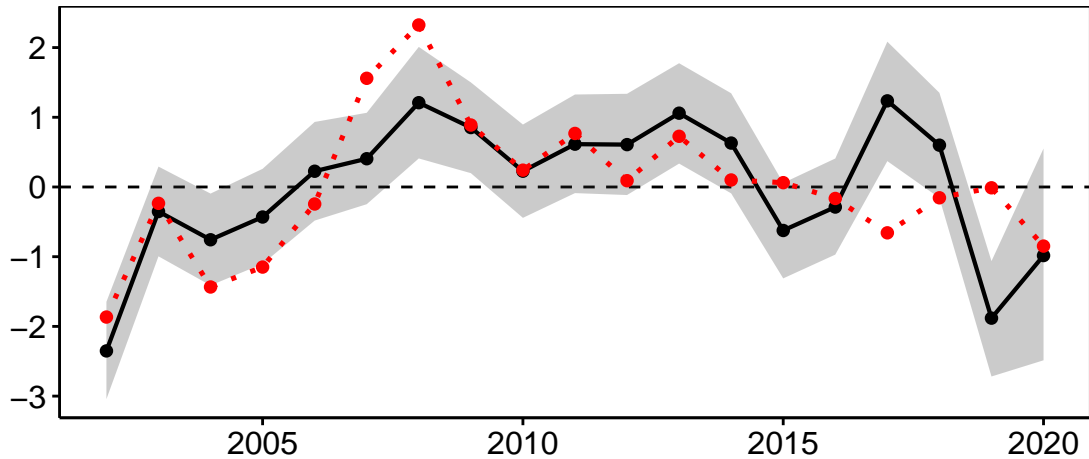

Supplement: Supplementary file 3 — Supplementary Software [file 41467_2021_26484_MOESM3_ESM.zip › Krill_SDM/figures/SDM_EPAC_predictions_vs_DeltaGLM_core.pdf]

# TSPIN

Standardized Index or CPUe

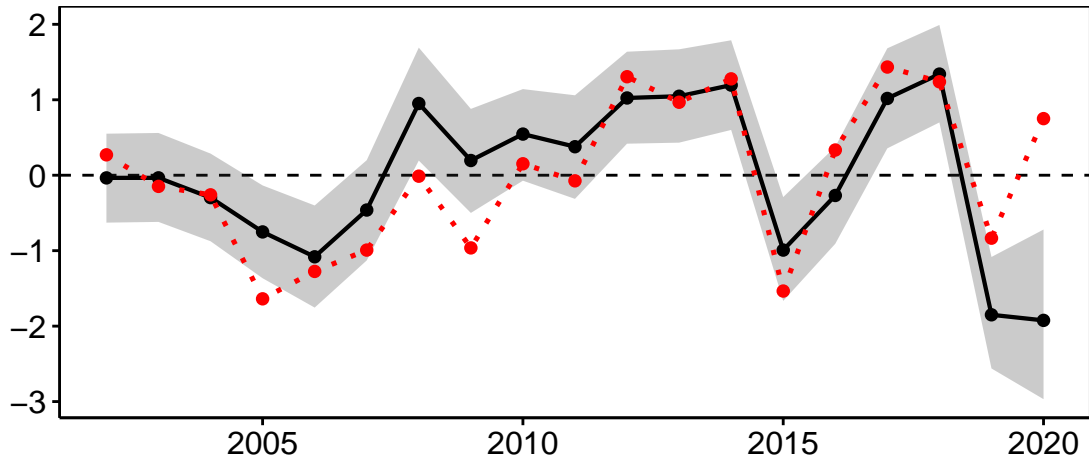

Supplement: Supplementary file 3 — Supplementary Software [file 41467_2021_26484_MOESM3_ESM.zip › Krill_SDM/figures/SDM_TSPIN_predictions_vs_DeltaGLM_core.pdf]
