# Supplementary material for: Diverse integrated ecosystem approach overcomes pandemic-related fisheries monitoring challenges
Source: Nat Commun. 2021 Nov 11;12:6492. doi: 10.1038/s41467-021-26484-5 (PMC8585921; doi:10.1038/s41467-021-26484-5)
Supplement: Supplementary file 4 — Reporting Summary [file 41467_2021_26484_MOESM4_ESM.pdf]

nature portfolio

Corresponding author(s): Jarrod A. Santora

Last updated by author(s): Aug 25, 2021

Reporting Summary

Nature Portfolio wishes to improve the reproducibility of the work that we publish. This form provides structure for consistency and transparency in reporting. For further information on Nature Portfolio policies, see our Editorial Policies and the Editorial Policy Checklist.

Statistics

For all statistical analyses, confirm that the following items are present in the figure legend, table legend, main text, or Methods section.

n/a	Confirmed	
☐	☒	The exact sample size (*n*) for each experimental group/condition, given as a discrete number and unit of measurement
☐	☒	A statement on whether measurements were taken from distinct samples or whether the same sample was measured repeatedly
☒	☐	The statistical test(s) used AND whether they are one- or two-sided<br>*Only common tests should be described solely by name; describe more complex techniques in the Methods section.*
☐	☒	A description of all covariates tested
☐	☒	A description of any assumptions or corrections, such as tests of normality and adjustment for multiple comparisons
☐	☒	A full description of the statistical parameters including central tendency (e.g. means) or other basic estimates (e.g. regression coefficient) AND variation (e.g. standard deviation) or associated estimates of uncertainty (e.g. confidence intervals)
☒	☐	For null hypothesis testing, the test statistic (e.g. *F*, *t*, *r*) with confidence intervals, effect sizes, degrees of freedom and *P* value noted<br>*Give P values as exact values whenever suitable.*
☐	☒	For Bayesian analysis, information on the choice of priors and Markov chain Monte Carlo settings
☐	☒	For hierarchical and complex designs, identification of the appropriate level for tests and full reporting of outcomes
☒	☐	Estimates of effect sizes (e.g. Cohen's *d*, Pearson's *r*), indicating how they were calculated

Our web collection on statistics for biologists contains articles on many of the points above.

Software and code

Policy information about availability of computer code

Data collection	No software was used for data collection. Observations were collected aboard a fisheries research vessel and then stored in a relational database, and transferred to an Open Access Server (NOAA ERRDAP Server: https://coastwatch.pfeg.noaa.gov/erddap/tabledap/FED_Rockfish_Catch.subset)
Data analysis	Data analysis was conducted using the statistical program Rstudio (version 3.5.3) and maps were prepared with ESRI ArcView (version 10). Models (delta-GLM, randomization/uncertainty estimation and krill species distribution models using boosted regression trees) were implemented in R (version 3.6.3). For the delta-GLM abundance estimation model, uncertainty in year effects was quantified by running the model in a Bayesian Framework with vague priors (4 chains and 5000 iterations) and computing 95% credible intervals using the package 'rstanarm'. Uncertainty bands are 95% credible intervals. Code for model estimation of forage species indicators and uncertainty is provided in the Source Code package. For seabird consumption models, standard regression models are used to examine predictions of fish abundance given the historical record as well as current metrics of seabird diet and trawl catch estimates.

For manuscripts utilizing custom algorithms or software that are central to the research but not yet described in published literature, software must be made available to editors and reviewers. We strongly encourage code deposition in a community repository (e.g. GitHub). See the Nature Portfolio guidelines for submitting code & software for further information.

Data

Policy information about availability of data

All manuscripts must include a data availability statement. This statement should provide the following information, where applicable:

- Accession codes, unique identifiers, or web links for publicly available datasets
- A description of any restrictions on data availability
- For clinical datasets or third party data, please ensure that the statement adheres to our policy

Data Availability:
The data generated in this study are provided in the Supplementary Information/Source Data file. The source data underlying Figures 1-4 and Supplementary Figures are provided.
All data pertaining to ecosystem indicators are available from the California Current Integrated Ecosystem Assessment: https://www.integratedecosystemassessment.noaa.gov/regions/california-current-region/index.html
All data from the RREAS is maintained on the NOAA ERDDAP portal and are freely accessible: NOAA Environmental Research Division Data Acquisition Portal (ERDDAP): https://coastwatch.pfeg.noaa.gov/erddap/tabledap/FED_Rockfish_Catch.html
Pacific Fisheries Management Council (PFMC) https://www.pcouncil.org/stock-assessments-star-reports-stat-reports-rebuilding-analyses-terms-of-reference/

Code Availability: Computer code, including model fitting, effort reduction simulation, and application of krill species distribution model, are provided within the Source Data Code file.

Field-specific reporting

Please select the one below that is the best fit for your research. If you are not sure, read the appropriate sections before making your selection.

- [ ] Life sciences
- [ ] Behavioural & social sciences
- [x] Ecological, evolutionary & environmental sciences

For a reference copy of the document with all sections, see nature.com/documents/nr-reporting-summary-flat.pdf

Ecological, evolutionary & environmental sciences study design

All studies must disclose on these points even when the disclosure is negative.

Study description	COVID-19 has drastically impacted ecosystem and fisheries monitoring data streams. To overcome these (data-poor) challenges, we apply a diverse integrated ecosystem approach to overcome obstacles for informing ecosystem state and health during the COVID-19 era. To maintain a nearly 40-year time series, we apply (1) new statistical models to address uncertainties of monitored fish species, (2) develop a novel simulation study using randomization to assess error in catch and improve survey design principles, (3) use diet observations of a sentinel seabird species along with historical catch data, to build new seabird indicator models, and (4) apply species distribution models that were trained on historical catch data to predict time series of relative abundance of krill species based on oceanographic conditions. Combined, these 'ecosystem tools' built on understanding of species intereactions, oceanclimate variability and catch statistics, offer a powerful framework for informing ecosystem conditions, especially during data-poor conditions as a result of COVID-19.
Research sample	There are 2 sample units: (1) a mid-water trawl sampling station event; used to summarize the 'catch-per-unit-effort' of species abundance (rockfish, anchovy, sanddabs, market squid, hake, octopus, krill) per trawl, and (2) an index of seabird (Common murre) prey consumption derived from observations of adult seabirds feeding young. Samples from (1) for krill species are used to train a species distribution model (SDM). Additional data on oceanographic conditions used in the SDM were derived from a dataassimilative (integrates satellite ocean conditions) oceanographic model that is publicly available.
Sampling strategy	Approximately 120 mid-water trawls are collected per survey and depending on weather, typically each sampling station or area is occupied 2-3 times per survey (standardized since 1983) both on and off the continental shelf. Sampling strategy and statistical power was assessed using a randomization procedure to simulate catch (relative abundance) and determine error as a funciton of number of trawls collected. Seabird diet data were collected using visual surveys (by an observer in a study blind) on Southeast Farallon Island; diet observations were collected daily following standardized protocols and the mean proportion of prey type was calculated as an index of prey availability.

Data collection	Fisheries data were collected aboard a research vessel by trained NOAA fishery biologists (a team of ~5-7 individuals working shifts; led by co-authors Sakuma and Field). All mid-water trawling was conducted at night following standardized protocols. Long-term (since 1983) sampling stations are fixed and located throughout the California Current Ecosystem. A team of fisheries scientists collected trawl data and identified all taxa immediately after each trawl. Data were entered into a relational database to assure data quality and assurance. During the COVID-19 era, the 2020 survey was heavily compromised due to ship cancellations and a limited number of samples were collected using a commercial vessel, thus providing a natural stress test to evaluate robustness of sample collections to inform ecosystem status.<br><br>Seabird diet data were collected using visual surveys (by an observer in a study blind suing binoculars; led by co-authors Warzybok and Jahncke) on Southeast Farallon Island; diet observations were collected daily following standardized protocols and the mean proportion of prey type was calculated as an index of prey availability. No seabirds were handled as part of this study.
Timing and spatial scale	The fisheries survey occurs each year from May-June and examines the mesoscale (1000s km) dynamics of biological and physical oceanography for the California Current Large Marine Ecosystem. The fishery survey typically samples May-June and conducts 30-45 days at sea for trawling operations. The survey domain spans 10 degrees of latitude (32N to 42N) covering the continental shelf (<200 m), slope (200-2000 m) and deeper waters (to 4000+ m), typically ranging 50 nautical miles offshore. The 2020 survey was restricted to the long-term core region to maintain the time series and 15 samples were collected during June 2020 over a period of 2 weeks. Southeast Farallon Island is one the biggest seabird colonies in the US West Coast. Field workers collect monitoring data throughout the year, but mostly focus on conditions during the breeding season (May-June) when adults are making frequent foraging trips to tend their young. Observations of seabird (common murre) prey are collected using visual survey techiques within a study blind and no animals were handled as part of this study. The seabird colony is located in the middle of the long-term mid-water trawl survey.
Data exclusions	No data were excluded from the analysis.
Reproducibility	All fisheries catch data are derived from observations and details are provided on how they were organized. The NOAA California Current Integrated Ecosystem Assessment stores all observation data and describes their reproducibility. All data underlying all figures are provided in the Source Data file.
Randomization	A simulation component of the study involves a novel re-sampling application of historical catch data. The analysis simulated 2020 sampling effort (COVID impacted) in past years to test whether the model (delta-GLM) could correct for sampling bias. A deterministic set of hauls was chosen each year (reflecting daily sampling lines) that best matched the 2020 effort to assess power and robustness of model estimates for target (monitoring) species.
Blinding	Not applicable to this study since it does not incorporate an experimental design or treatments (i.e., this is an empirical study applying modeling techniques).

Did the study involve field work? ☒ Yes ☐ No

Field work, collection and transport

Field conditions	Data are typically collected on a NOAA Fisheries Survey Vessel (FSV) during the annual NOAA-NMFS Rockfish Recruitment and Ecosystem Assessment Survey. Due to suspension of most NOAA vessel research surveys in early 2020 in response to the COVID 19 epidemic, data for the 2020 sampling year were collected from a chartered commercial fishing vessel with experience deploying trawl gear. Field conditions during the spring upwelling season in the California Current are typically associated with persistent NW winds.
Location	California Current Large Marine Ecosystem. The survey domain spans 10 degrees of latitude (32N to 42N) covering the continental shelf (<200 m), slope (200-2000 m) and deeper waters (to 4000+ m), typically ranging 50 nautical miles offshore.
Access & import/export	All samples are collected from U.S. Exclusive Economic Zone (EEZ) marine waters (typically within 2-50 miles of the shoreline) on government or government chartered research vessels. All sampling is done in accordance with state and federal sampling permits. Samples are typically processed at sea, however in 2020 catch data were subsampled at sea and transported to land for enumeration in a NOAA laboratory facility.
Disturbance	All sampling is done in accordance with state and federal sampling permits. These include permits issued under the marine mammal protection act, for which disturbance and risk to marine mammals is mitigated by conducting marine mammal watches prior to and during all trawling operations, with trawling halted if mammals are observed within 1 nautical mile of sampling locations.

Reporting for specific materials, systems and methods

We require information from authors about some types of materials, experimental systems and methods used in many studies. Here, indicate whether each material, system or method listed is relevant to your study. If you are not sure if a list item applies to your research, read the appropriate section before selecting a response.

Materials & experimental systems

n/a	Involved in the study
☒	☐ Antibodies
☒	☐ Eukaryotic cell lines
☒	☐ Palaeontology and archaeology
☐	☒ Animals and other organisms
☒	☐ Human research participants
☒	☐ Clinical data
☒	☐ Dual use research of concern

Methods

n/a	Involved in the study
☒	☐ ChIP-seq
☒	☐ Flow cytometry
☒	☐ MRI-based neuroimaging

Animals and other organisms

Policy information about studies involving animals; ARRIVE guidelines recommended for reporting animal research

Laboratory animals	No laboratory animals are used in this study.
Wild animals	The midwater trawl survey requires the take of small fish and invertebrate species (micronekton, typically smaller than 300mm). These include euphausiids and other epipelagic crustaceans, young-of-the-year rockfish (Sebastes) and other groundfish, multiple ontogenetic stages of anchovies, sardines and other pelagic forage fish, pelagic squids, and various other pelagic vertebrates and invertebrates. All takes are authorized under relevant state and federal permits.
Field-collected samples	Most organisms do not survive sampling in trawls, and most organisms are returned to the sea following taxonomic identification, enumeration and measurement. Limited numbers of organisms or tissue samples are returned to laboratories for various analyses. In 2020, due to COVID 19, subsamples of catches were transported to the laboratory for identification, enumeration and additional analyses.
Ethics oversight	No ethical approval is needed for collecting samples of micronekton (planktonic) organisms for fishery investigations. All fisheries research is monitored by the U.S. government.

Note that full information on the approval of the study protocol must also be provided in the manuscript.
